# Association between admission undernutrition and mortality rates and adverse outcomes in a pediatric intensive care unit in India: a prospective cohort study

**DOI:** 10.3389/fped.2026.1880294

**Published:** 2026-08-17

**Authors:** Vijay Raghunath Kalrao, Aastha Pramod

**Affiliations:** Department of Pediatrics, Bharati Vidyapeeth (Deemed to be University) Medical College, Pune, Maharashtra, India

**Keywords:** child, India, low- and middle-income countries, malnutrition, pediatric intensive care, propensity score, severity-of-illness score

## Abstract

**Background:**

Undernutrition is common in critically ill children in resource-limited settings. However, it remains unclear whether its association with pediatric intensive care unit (PICU) outcomes persists after rigorous adjustment for illness severity, particularly with the use of mortality prediction scores developed in high-income populations. Therefore, in this study, we examined whether undernutrition at admission was associated with mortality and adverse outcomes in an Indian tertiary care PICU.

**Methods:**

We conducted a prospective cohort study between August 2022 and June 2024 in a 16-bed mixed medical-surgical PICU at a tertiary university hospital in India. The study included 331 critically ill children aged 1 month to 18 years with complete data. Undernutrition was defined using the World Health Organization and Indian Academy of Pediatrics growth standards (z-score < −2 SD) measured within 24 h of admission. The primary outcome was PICU mortality rate. Adjusted risk ratios (aRRs) were estimated using modified Poisson regression, adjusting for Pediatric Index of Mortality (PIM-3)-predicted mortality, sepsis, age, and chronic comorbidities.

**Results:**

At admission, 60 (18.1%) children were undernourished. The PICU mortality rate was 13.9% overall, 30.0% among undernourished children, and 10.3% among well-nourished children. After multivariable adjustment, undernutrition was associated with a higher mortality risk [aRR 2.78, 95% confidence interval (CI) 1.58–4.91], corresponding to an adjusted absolute risk difference of 18.6 percentage points. The association persisted in the propensity score–weighted analysis (aRR 2.59, 95% CI 1.34–5.03) but was attenuated when extreme illness severity was excluded from the analysis. Undernutrition was also associated with mechanical ventilation (66.7% vs. 35.4%; aRR 1.84, 95% CI 1.40–2.41), extended PICU stay (61.7% vs. 37.0%; aRR 1.56, 95% CI 1.17–2.09), and the death-or-mechanical ventilation composite (66.7% vs. 36.2%; aRR 1.76, 95% CI 1.35–2.30) in full-cohort analyses designed to avoid survivor-restriction bias. The observed mortality substantially exceeded the PIM-3 predictions (standardized mortality ratio, 4.63), particularly in undernourished children (standardized mortality ratio, 7.90).

**Conclusion:**

In this Indian cohort, undernutrition at admission was associated with higher PICU mortality and adverse outcomes after adjusting for illness severity. In high-burden, resource-limited settings, anthropometric screening may aid risk stratification. However, causal inference remains limited by the observational design and substantial miscalibration of the illness severity score.

## 1 Introduction

In low- and middle-income countries (LMIC), undernutrition contributes to nearly 50% of all deaths in children aged <5 years (1–3). In India, more than one-third of children aged <5 years are stunted or underweight (4, 5). Recent meta-analyses indicate that over one-third of pediatric intensive care unit (PICU) admissions in LMIC involve undernourished children (6, 7). Undernutrition compromises immunity, impairs barrier function, reduces respiratory muscle strength, and depletes metabolic reserves (8, 9). These preexisting vulnerabilities, combined with metabolic stress and resource constraints, can increase the risk of complications and death (10, 11). Despite this biological plausibility, the evidence linking undernutrition to PICU outcomes remains heterogeneous. Some studies have reported inconsistent effect sizes, which likely reflect variations in nutritional definitions, adequacy of severity adjustment, and population characteristics (12–14). Widely used pediatric mortality prediction values, including the Pediatric Index of Mortality (PIM-3) score, do not incorporate anthropometric data into their calculations. It remains unclear whether undernutrition at admission provides prognostic information beyond the PIM-3 score in high-burden, resource-limited settings (15, 16). Most prior evidence is derived from high-income settings or relies on retrospective designs with variable anthropometric assessment quality and illness severity adjustment (12, 13). We hypothesized that undernutrition is associated with higher PICU mortality and worse secondary outcomes after adjusting for confounders.

Therefore, this prospective cohort study aimed to evaluate whether anthropometrically defined undernutrition at PICU admission was associated with mortality rates and adverse outcomes after adjusting for PIM-3-based illness severity in a tertiary PICU in India.

## 2 Materials and methods

### 2.1 Study design and setting

This prospective cohort study was conducted between August 2022 and June 2024 in the PICU of a tertiary care university hospital in India. We followed the Strengthening the Reporting of Observational Studies in Epidemiology guidelines (17). The University Medical College Institutional Ethics Committee (DHR Reg. No. EC/NEW/INST/2020/656) reviewed and approved this study (Ref: BVDUMC/IEC/38, 11 August 2022). Informed consent was obtained from the guardians of the participants of the study. All procedures were performed in accordance with the ethical standards of the Institutional Ethics Committee and the 1964 Declaration of Helsinki and its later amendments.

Our hospital's PICU is a 16-bed mixed medical-surgical unit that admits approximately 350–400 patients annually, with a population comprising 80% medical and 20% surgical, trauma, or toxicology cases from lower-middle-income urban and rural backgrounds.

### 2.2 Participants and data collection

All consecutive admissions of children aged 1 month to 18 years were screened for inclusion criteria. Trained research personnel prospectively gathered demographic, clinical, and outcome data using a standardized case report form until the patients were discharged from the PICU or until their death. Data were recorded before outcome ascertainment to minimize bias. We excluded children with conditions precluding reliable anthropometry (e.g., gross edema and severe skeletal anomalies), transfer or discharge within 24 h, and readmission during the same hospitalization. The analytical cohort comprised unique index admissions with complete anthropometric, outcome, and covariate data points.

### 2.3 Exposure and outcome definitions

The primary exposure was undernutrition [defined as a z-score < −2 standard deviation (SD)] vs. well-nourished status (defined as a z-score ≥ −2 SD) with the use of the World Health Organization weight-for-height or length standards for children aged ≤5 years (18) or the Indian Academy of Pediatrics body mass index-for-age standards for children aged >5 years (19). We obtained anthropometric measurements (weight, length, and height) within 24 h of PICU admission using calibrated equipment. When acute measurement was not feasible because of hemodynamic instability, the most recent reliable measurement from the preceding seven days was used.

The primary outcome was PICU mortality. Secondary outcomes included mechanical ventilation requirements, extended PICU stay (>3 days), healthcare-associated infections (20), multiorgan dysfunction syndrome, vasoactive infusion requirements, and ventilator-free days until day 28.

Healthcare-associated infections were prospectively and actively surveilled throughout the PICU stay by trained research personnel using standardized Centers for Disease Control and Prevention (CDC)/National Healthcare Safety Network (NHSN) surveillance definitions (20). Surveillance was continuously applied from admission using clinical, microbiological, and radiological data collected prospectively, independent of the routine clinical documentation.

multiple organ dysfunction syndrome was defined as the concurrent dysfunction of two or more organ systems based on the pediatric organ dysfunction criteria from the International Pediatric Sepsis Consensus Conference (21).

### 2.4 Covariates

Covariates for multivariate adjustment were selected *a priori* based on clinical relevance and prior literature (12–15), without reference to their association with outcomes in this dataset. The prespecified covariates included age (continuous, per 1-year increase), PIM-3-predicted mortality values (continuous, per 1-standard deviation increase), sepsis at admission (binary), and chronic comorbidities (binary).

Sepsis at admission was operationally defined by the treating physician using a pediatric adaptation of the Sepsis-3 framework as suspected or confirmed infection present at admission with an acute increase in pSOFA score ≥2 points from baseline (baseline was assumed to be zero in patients without known preexisting organ dysfunction), consistent with the approach described by Matics and Sanchez-Pinto (22).

Illness severity at PICU admission was quantified using the PIM-3 score, which was calculated according to the original algorithm (23). The PIM-3 score was used to predict mortality (model-estimated probability of death at admission, expressed as a percentage) and entered as a continuous variable in multivariable models.

### 2.5 Assessment of multicollinearity

Multicollinearity was evaluated among the covariates using Pearson's correlation coefficients for continuous variables (age and PIM-3-predicted mortality) and variance inflation factors from the primary multivariable logistic regression model.

### 2.6 Missing data

We performed complete case analyses for all outcomes. Children with missing outcomes or covariate data required for a given model were excluded from the analyses, and no data imputation was performed. Missing data were minimal; no patient had missing covariates for the primary outcome analysis, only one patient had a missing multiorgan dysfunction syndrome classification, and one patient had missing data for an extended length of stay in the PICU.

### 2.7 Statistical analysis

The sample size was estimated *a priori*, assuming a 19% prevalence of undernutrition (4), mortality rates of 16% in well-nourished children and 45% in undernourished children (24), and a two-sided alpha of 0.05, providing 90% power with 185 participants. We prospectively enrolled 331 participants, which exceeded the minimum required sample size, during the study period.

#### 2.7.1 Primary outcome analysis

Baseline characteristics were compared across nutritional groups using the chi-square test for categorical variables and Wilcoxon rank-sum test for continuous variables. To estimate the association between undernutrition and PICU mortality, we used a modified Poisson regression with a log link and robust standard error (25). The primary multivariate model was adjusted for age, PIM-3-predicted mortality, sepsis at admission, and chronic comorbidities. For binary undernutritional exposure, adjusted absolute risk differences and numbers needed to harm (NNH) were estimated using marginal standardization (g-computation) with 95% confidence intervals (CIs) from 1,000 non-parametric bootstrap resamples using the percentile method.

#### 2.7.2 Model discrimination and calibration

The event-per-variable ratio was 9.2 (46 events and five predictors). Model discrimination and calibration were assessed using a prespecified logistic regression model with the same set of predictors. This model was used only for validation, and the primary inference regarding the risk ratios was based on the modified Poisson regression. The apparent performance was calculated for the full cohort, with discrimination summarized using the area under the receiver operating characteristic curve (AUC). Calibration was assessed using calibration-in-the-large (intercept) and calibration slope, supplemented with decile-based calibration plots. Internal validation was performed using 1,000 bootstrap resampling iterations for each cohort. For each resampling, ridge-penalized logistic regression was used to mitigate the quasi-complete separation that can occur with sparse events.

#### 2.7.3 Secondary outcomes

Secondary outcomes were evaluated using modified Poisson regression with robust standard errors for binary outcomes and linear regression for ventilator-free days up to 28 days after admission. The models were adjusted for variables, including age, baseline illness severity as indicated by the PIM-3 predicted mortality, chronic or comorbid conditions, and sepsis at admission. Given that death may prevent the observation of extended PICU stays or prolonged mechanical ventilation, analyses limited to survivors were excluded from the main manuscript. Instead, the analysis encompassed the entire cohort and composite outcomes, including mortality. For the extended PICU stay outcome in the full cohort (non-composite), the duration was determined by each child's actual observed PICU stay, including those who died, rather than using imputed or artificially assigned values. Adjusted absolute risk differences were estimated using marginal standardization (g-computation), with 95% confidence intervals obtained from 2,000 non-parametric bootstrap resamples. The corresponding numbers needed to harm were derived when the adjusted risk difference confidence interval excluded zero for the binary secondary outcomes. The *P*-values for the secondary outcomes were adjusted using the Benjamini–Hochberg procedure to control the false discovery rate. As a sensitivity analysis for the length-of-stay process, we fitted a cause-specific Cox proportional hazards model for time to alive PICU discharge, treating death as a competing event.

#### 2.7.4 Sensitivity and exploratory analyses

We performed prespecified sensitivity analyses to assess the robustness of the primary association, including propensity score-weighted doubly robust estimation, exclusion of extreme PIM-3 values, winsorization of PIM-3, exclusion of obese children, and alternative modeling of illness severity.

In response to methodological concerns raised during the peer review, we conducted four additional analyses not included in the original protocol: age-stratified mortality analyses with formal interaction testing (linear, categorical, and spline-based terms), repeated within the propensity-weighted framework; a E-value analysis to quantify sensitivity to unmeasured confounding; net reclassification improvement (NRI) and discrimination (AUC) to assess whether undernutrition improved individual-level risk classification beyond the base clinical model; and median regression as a sensitivity analysis for ventilator-free days to day 28, which showed a marked ceiling effect.

In addition, we conducted prespecified exploratory analyses of effect modification by illness severity, sepsis-stratified associations, standardized mortality ratios, dose–response across nutritional severity, and analyses excluding trauma or toxicology admissions. These were not adjusted for multiple comparisons and should be interpreted as hypothesis-generating.

The full methods for the sensitivity and exploratory analyses are provided in the Supplementary Material.

#### 2.7.5 Statistical software

Statistical analyses were conducted using *R* version 4.4.3 with the dplyr (1.1.4), readxl (1.4.3), sandwich (3.1-0), boot (1.3-30), pROC (1.18.5), glmnet (4.1-8), WeightIt (1.2.0), cobalt (4.5.5), logistf (1.26.0), car (3.1-2), and survival (3.8-6) packages.

## 3 Results

### 3.1 Study population

**Figure 1:**
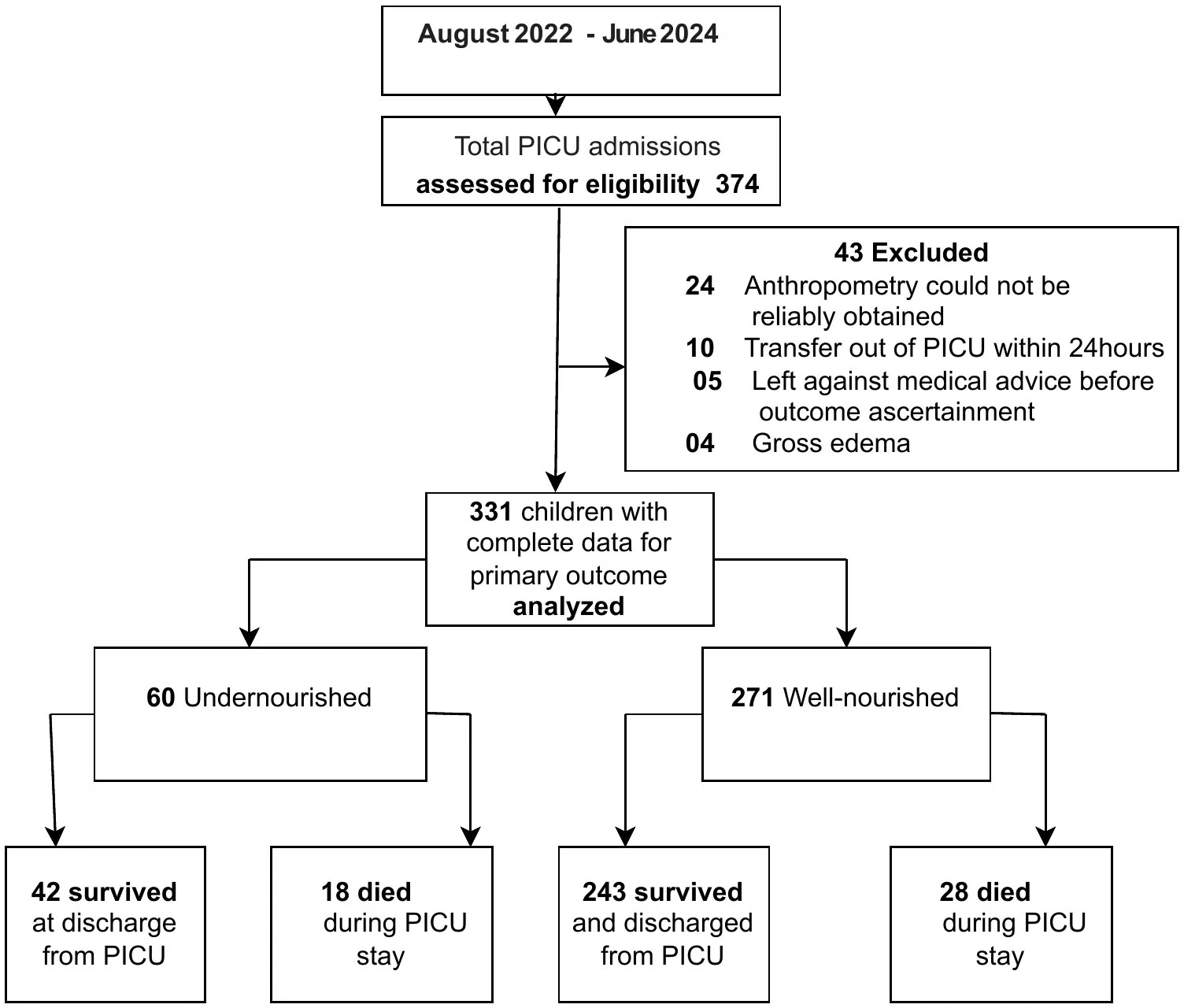
A participant flow diagram of the study. This figure documents the screening, exclusion, and enrollment processes in accordance with the STROBE reporting guidelines for cohort studies. During the 22-month study period (August 2022 to June 2024), 374 consecutive pediatric intensive care unit (PICU) index admissions were screened for their eligibility. Readmissions during the same hospitalization period were excluded. Of the 374 screened admissions, 43 (11.5%) patients were excluded, comprising 24 children in whom reliable anthropometry could not be obtained for reasons other than edema, 4 children in whom gross edema precluded anthropometric assessment, 10 children who were transferred or discharged within 24 h, and 5 children who were discharged against medical advice. The final analytical cohort comprised 331 children with complete anthropometric and outcome data.

During the study period, 374 children were admitted to the PICU. Forty-three (11.5%) patients were excluded because reliable anthropometry could not be obtained (*n* = 24), patients were transferred or discharged within 24 h (*n* = 10), patients left the hospital against medical advice before outcome ascertainment (*n* = 5), or gross edema precluded anthropometric assessment (*n* = 4), leaving 331 unique index admissions (Figure 1). Among the 28 children excluded for anthropometric reasons, baseline illness severity and outcome data were available. The median PIM-3 predicted mortality in these children was 1.5% (IQR, 0.1%–2.9%), and the observed mortality was 3/28 (10.7%).

At admission, 60 of the 331 children (18.1%) were undernourished and 271 (81.9%) were well-nourished. Obesity was uncommon (7 children, 2.1%). Compared with well-nourished children, undernourished children were younger [median age, 1.50 years [IQR, 0.81–4.04] vs. 5.08 years [IQR, 1.71–10.84]; *P* < 0.001] and more frequently had sepsis at admission (76.7% vs. 44.3%; *P* < 0.001). PIM-3 predicted mortality was similar between the two groups [median, 1.60% [IQR, 0.10%–7.60%] vs. 1.30% [IQR, 0.10%–3.80%]; *P* = 0.092], as were female sex (35.0% vs. 43.2%; *P* = 0.245), maternal education (*P* = 0.123), and chronic comorbid conditions (33.3% vs. 25.8%; *P* = 0.237). Disease category and primary organ system involvement differed according to nutritional status (Table 1). Chronic comorbidity categories by organ system, prevalence of anthropometric deficits by age group, and anthropometric profiles of undernourished vs. well-nourished children are presented in Supplementary Tables S1a–c.

**Table 1:** Baseline characteristics, clinical profile, and outcomes in children according to nutritional status.

Characteristic (*n*)	Overall (331)	Undernourished (60)	Well-nourished (271)	*P*-value
Demographics^a^				
Age, years, median (IQR)	4.08 (1.33–10.04)	1.50 (0.81–4.04)	5.08 (1.71–10.84)	<0.001
Female sex, No. (%)	138 (41.7)	21 (35.0)	117 (43.2)	0.245
Maternal education, No. (%)				0.123
Illiterate to middle school	77 (23.3)	9 (15.0)	68 (25.1)	
High school to higher secondary	157 (47.4)	28 (46.7)	129 (47.6)	
Graduate or postgraduate	97 (29.3)	23 (38.3)	74 (27.3)	
Illness severity and clinical characteristics				
PIM-3-predicted mortality at admission, %, median (IQR)	1.30 (0.10–5.20)	1.60 (0.10–7.60)	1.30 (0.10–3.80)	0.092
Sepsis at admission, *n* (%)	166 (50.2)	46 (76.7)	120 (44.3)	<0.001
Disease category, n (%)				<0.005
Medical	263 (79.5)	54 (90.0)	209 (77.1)	
Surgical	9 (2.7)	3 (5.0)	6 (2.2)	
Trauma/toxicology	59 (17.8)	3 (5.0)	56 (20.7)	
Comorbid condition present, n (%)	90 (27.2)	20 (33.3)	70 (25.8)	0.237
Primary organ system involved, n(%)				<0.001
Pulmonary	103 (31.1)	29 (48.3)	74 (27.3)	
CNS	73 (22.1)	5 (8.3)	68 (25.1)	
Gastrointestinal	24 (7.3)	4 (6.7)	20 (7.4)	
Cardiac	11 (3.3)	2 (3.3)	9 (3.3)	
Haematology-oncology	32 (9.7)	10 (16.7)	22 (8.1)	
Others	88 (26.6)	10 (16.7)	78 (28.8)	
Outcomes during PICU stay^b^				
MODS during PICU, No. (%)^c^	64 (19.3)	25 (41.7)	39 (14.4)	<0.001
Mortality, No. (%)	46 (13.9)	18 (30.0)	28 (10.3)	<0.001
Mechanical ventilation required, No. (%)	136 (41.1)	40 (66.7)	96 (35.4)	<0.001
Extended PICU stay >3 days, No. (%)^d^	137 (41.4)	37 (61.7)	100 (37.0)	<0.001
Hospital-acquired infection, No. (%)	54 (16.3)	21 (35.0)	33 (12.2)	<0.001
Vasoactive infusion required, No. (%)	78 (23.6)	25 (41.7)	53 (19.6)	<0.001
VFD-28 in all children, days, median (IQR)^c^	28.00 (24.00–28.00)	23.50 (0.00–28.00)	28.00 (26.00–28.00)	<0.001

CNS, central nervous system; IQR, interquartile range; MODS, multiple organ dysfunction syndrome; PICU, pediatric intensive care unit; PIM-3, Pediatric Index of Mortality-3; VFD28, ventilator-free days at 28 days.

Undernutrition was defined as a weight-for-height/length or body mass index-for-age z-score <−2SD using the World Health Organization growth standards (children aged ≤5 years) or the Indian Academy of Pediatrics growth standards (children aged >5 years). The PIM-3 predicted mortality at admission represents the model-estimated probability of death at PICU admission, expressed as a percentage, with higher values indicating greater risk.

a Continuous variables were presented as medians (IQR) and were compared using the Wilcoxon rank-sum test. Categorical variables were presented as numbers and percentages (%) and were compared using the *χ*^2^-test. Statistical significance was set at *P* < 0.05.

b Outcomes were presented using the full cohort rather than restricting the analyses to survivors.

c VFD28 in all children was assigned as 0 for children who died, 28 for survivors who did not require mechanical ventilation, 28 minus days of mechanical ventilation for those ventilated for ≤28 days, and 0 for those ventilated for >28 days.

d One patient each had missing data for MODS classification and Length of PICU stay and were excluded from the respective analyses.

In crude full-cohort analyses, undernourished children experienced worse outcomes than well-nourished children. Mortality was higher in undernourished children [30.0% [18/60] vs. 10.3% [28/271]; *P* < 0.001], as were the frequencies of mechanical ventilation (66.7% vs. 35.4%; *P* < 0.001), PICU stay longer than 3 days (61.7% vs. 37.0%; *P* < 0.001), healthcare-associated infection (35.0% vs. 12.2%; *P* < 0.001), vasoactive infusion requirement (41.7% vs. 19.6%; *P* < 0.001), and multiorgan dysfunction syndrome (41.7% vs. 14.4%; *P* < 0.001). Ventilator-free days at day 28 were fewer among undernourished children [median, 23.5 [IQR, 0–28] vs. 28.0 [IQR, 26–28] days; *P* < 0.001]. The overall PICU mortality rate was 13.9% (46/331) **(**Table 1).

### 3.2 Primary outcome

Before fitting the primary model, we performed a multicollinearity assessment that confirmed model stability, with a negligible correlation between age and PIM-3 predicted mortality (*r* = 0.02), and all variance inflation factors were below 1.25, indicating no meaningful multicollinearity among predictors.

Crude PICU mortality was higher among undernourished children than among well-nourished children [30.0% [18/60] vs. 10.3% [28/271]; crude risk ratio (RR), 2.90; 95% CI 1.72–4.89; *P* < 0.001]. In the prespecified multivariable model adjusted for age, PIM-3 predicted mortality, sepsis at admission, and chronic comorbid conditions, undernutrition was associated with higher mortality (aRR, 2.78; 95% CI 1.58–4.91; *P* < 0.001; Table 2). This corresponded to an adjusted absolute risk difference of 18.6 percentage points (95% CI 1.2–35.4), indicating approximately one additional death for every five undernourished children compared with well-nourished children (NNH, 5; 95% CI 3–26). Given the small number of events (*n* = 46), this estimate was imprecise and should be interpreted in the context of the substantial PIM-3 miscalibration in this cohort [standardized mortality ratio (SMR) 4.63], which raises the possibility of residual confounding by illness severity (Section 4.7).

**Table 2:** Baseline covariates associated with PICU mortality.

Variable	Non-survivor, n/total (%)	Crude RR (95% CI)	*P-*value	Adjusted RR^a^ (95% CI)	*P*-value adjusted	Adjusted ARD^b^ (95% CI)^c^	NNH^b^ (95% CI)^c^
Age, per 1-year increase		1.01 (0.96–1.07)	0.696	1.06 (1.00–1.12)	0.038		
Chronic/comorbid condition							
Absent	34/241 (14.1%)	1 [Reference]		1 [Reference]			
Present	12/90 (13.3%)	0.95 (0.51–1.74)	0.856	0.87 (0.52–1.44)	0.580		
Nutritional status							
Well-nourished	28/271 (10.3%)	1 [Reference]		1 [Reference]			
Undernourished	18/60 (30.0%)	2.90 (1.72–4.89)	0.001	2.78 (1.58–4.91)	<0.001	18.6 (1.2–35.4)	5 (3–26)
PIM-3 predicted mortality at admission (per 1-SD increase)		1.27 (1.15–1.41)	<.001	1.39 (1.25–1.54)	<0.001		
Sepsis at admission							
No	11/165 (6.7%)	1 [Reference]		1 [Reference]			
Yes	35/166 (21.1%)	3.16 (1.66–6.01)	<0.001	3.57 (1.60–7.96)	0.002	16.1 (5.7–30.1)	6 (3–18)

ARD, absolute risk difference; CI, confidence interval; NNH, number needed to harm; PICU, pediatric intensive care unit; PIM-3, Pediatric Index of Mortality-3; RR, risk ratio; SD, standard deviation.

Chronic or comorbid conditions and sepsis at admission were coded as present or absent (reference). Sepsis was defined as a clinical diagnosis prior to PICU admission. PIM-3 predicted mortality at admission represented the model-estimated probability of death, expressed as a percentage entered per 1-SD increase in the adjusted model.

a Adjusted RRs were estimated from a prespecified modified Poisson regression model with a log link and robust standard error. Continuous predictors were expressed per 1-unit increase, unless otherwise specified; the PIM-3 predicted mortality was entered per 1-SD increase.

b Adjusted ARDs and NNH were estimated using marginal standardization (g-computation) and were presented only for binary baseline exposures (nutritional status and sepsis upon admission).

c 95% CIs for ARDs and NNH were obtained from 1,000 non-parametric bootstrap resamples (percentile method).

Sepsis at admission was also independently associated with mortality (aRR, 3.57; 95% CI 1.60–7.96; *P* = 0.002). Each 1-SD increase in PIM-3 predicted mortality was associated with a 39% higher mortality risk (aRR, 1.39; 95% CI 1.25–1.54; *P* < 0.001). Age showed a modest association (aRR per year, 1.06; 95% CI 1.00–1.12; *P* = 0.038), whereas chronic comorbid conditions were not independently associated with mortality.

### 3.3 Model performance

**Figure 2:**
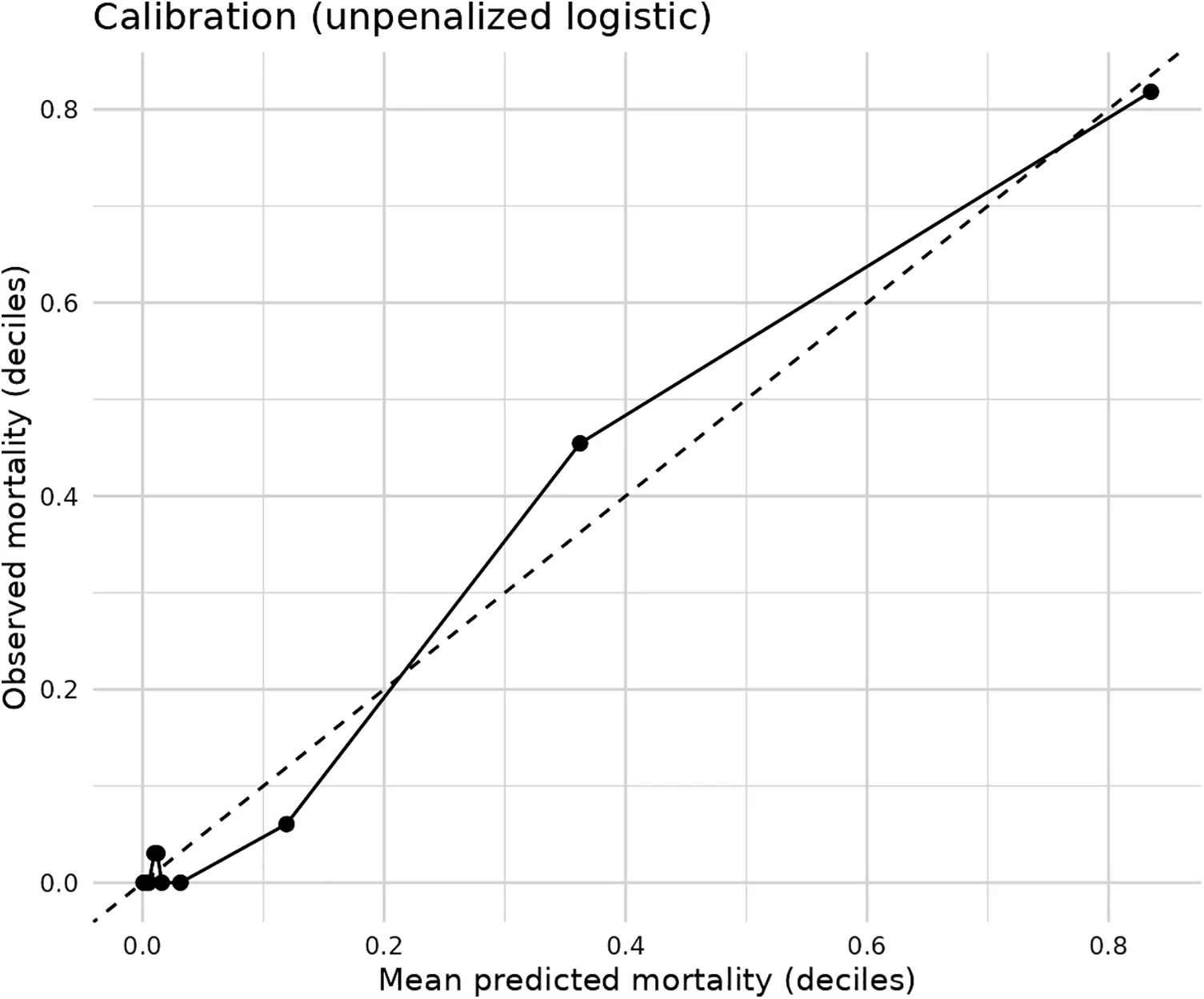
Calibration of the prespecified PICU mortality prediction model (standard unpenalized logistic regression). Patients were grouped into deciles according to their predicted mortality risk. Within each decile, the observed proportion of deaths (*y*-axis) was plotted against the mean predicted probability of death (*x*-axis). The dashed diagonal line represents the perfect calibration. The model showed good overall calibration across the lower and middle risk spectra, with some underestimation of mortality at the highest predicted risk decile, consistent with the PIM-3 miscalibration in this setting. A small number of fitted probabilities approached 1.0, which was consistent with quasi-complete separation in sparse covariate patterns. This motivated ridge penalization during bootstrap internal validation (Supplementary Figure S2).

The mortality prediction model demonstrated high apparent discrimination (AUC 0.952) and an optimism-corrected AUC of 0.946 after bootstrap internal validation; however, both values should be interpreted cautiously, given the limited event count (*n* = 46, EPV 9.2), which creates conditions favorable for optimism, even with bootstrap correction (Table 3). The apparent calibration slope was 1.000, and the optimism-corrected calibration slope was 0.855, indicating modest overfitting, which is typical of models developed with limited events. Bootstrap internal validation confirmed a consistent elevation of the undernutrition–mortality association across resamples (Supplementary Table S1a). Together, the calibration plots and risk distribution indicated that model discrimination and calibration were not driven by a small number of near-deterministic predictions and that the validation results were robust to ridge penalization (Figure 2; Supplementary Figures S1, S2).

**Table 3:** Internal validation for the primary PICU mortality model.

Metric	Parameter	Value
Events per variable		9.2
Adjusted RR^a^ for undernutrition (modified Poisson, robust SE)		2.78 (95% CI 1.58–4.91)
Apparent AUC^b^ (c-statistic) from standard logistic model		0.952
Optimism-corrected^c^ (bootstrap refits use ridge-penalized logistic)		0.946
Calibration slope^b^ (apparent)		1.000
Optimism-corrected calibration slope^d^ (bootstrap refits use ridge-penalized logistic)		0.855
Predicted risk (standard logistic)	Minimum	0.001
	1st Percentile	0.001
	99th Percentile	0.984
	Maximum	1.000

AUC, area under the receiver operating characteristic curve; CI, confidence interval; RR, relative risk; SE, standard error.

a The logistic regression model shown here was used only for calibration assessment; primary inference (adjusted RR) was based on the modified Poisson regression, as specified.

b Apparent AUC and apparent calibration slope were obtained from the unpenalized logistic model.

c Optimism-corrected AUC was obtained from bootstrap refitting with L2-penalized (ridge) logistic regression to mitigate quasi-complete separation.

d The optimism-corrected calibration slope of 0.855 reflects modest overfitting, which is typical in model development with limited events and moderately extreme tail predictions.

Calibration plots are provided in Figure 2 and Supplementary Figure S2; the predicted-risk distribution is provided in Supplementary Figure S1.

### 3.4 Secondary outcomes

Undernutrition was associated with a higher adjusted risk of all prespecified secondary outcomes (Table 4). To reduce survivor-restriction bias for length-of-stay and ventilation outcomes, we analyzed these outcomes in the full cohort and constructed composite endpoints incorporating death: adjusted RRs were 1.56 (95% CI 1.17–2.09) for extended PICU stay longer than 3 days (61.7% vs. 37.0%), 1.57 (95% CI 1.24–1.99) for death or extended PICU stay (75.0% vs. 43.2%), 1.84 (95% CI 1.40–2.41) for mechanical ventilation in the full cohort (66.7% vs. 35.4%), and 1.76 (95% CI 1.35–2.30) for death or mechanical ventilation (66.7% vs. 36.2%).

**Table 4:** Secondary outcomes according to nutritional status.

Outcome	Well nourished (%)	Undernourished (%)	Adjusted RR^a^/mean difference (95% CI)	*P*-value (BH-adjusted)	ARD^b^ (95% CI)^c^	NNH^b^ (95% CI)^c^
Extended PICU stay (>3 days), full cohort^d^	100/270 (37.0)	37/60 (61.7)	1.56 (1.17–2.09)	0.003	24.7 (8.9–40.5)	4 (2–11)
Death or extended PICU stay (>3 days), full cohort	117/271 (43.2)	45/60 (75.0)	1.57 (1.24–1.99)	<0.001	26.9 (12.5–41.3)	4 (2–8)
Mechanical ventilation required, full cohort	96/271 (35.4)	40/60 (66.7)	1.84 (1.40–2.41)	<0.001	31.3 (16.3–46.3)	3 (2–6)
Death or mechanical ventilation, full cohort	98/271 (36.2)	40/60 (66.7)	1.76 (1.35–2.30)	<0.001	30.5 (15.6–45.4)	3 (2–6)
Ventilator-free days to day 28, full cohort	28.0 (26.0–28.0)	23.5 (0.0–28.0)	−3.8 (−6.1 to −1.4)	0.004		
Healthcare-associated infection (HAI)	33/271 (12.2)	21/60 (35.0)	1.93 (1.19–3.14)	0.014	17.3 (6.2–29.3)	6 (4–17)
MODS during PICU stay^d^	39/270 (14.4)	25/60 (41.7)	2.55 (1.60–4.05)	<0.001	25.1 (12.5–37.7)	4 (3–8)
Vasoactive infusion required	53/271 (19.6)	25/60 (41.7)	1.51 (1.06–2.17)	0.035	12.0 (1.2–23.3)	8 (4–87)

ARD, absolute risk difference; BH, Benjamini–Hochberg; CI, confidence interval; HAI, healthcare-associated infection; IQR, interquartile range; MODS, multiple organ dysfunction syndrome; NNH, number needed to harm; PICU, pediatric intensive care unit; RR, risk ratio.

Data were presented as *n* (%) or medians (IQR).

Undernutrition was defined as weight-for-height/length or body mass index-for-age z-score <−2 SD using the World Health Organization growth standards (children ≤5 years) or Indian Academy of Pediatrics growth standards (children >5 years).

a Adjusted RRs and mean differences were estimated using modified Poisson regression with robust standard errors for binary outcomes and linear regression for ventilator-free days, adjusted for age, PIM-3 predicted mortality at admission, chronic/comorbid conditions, and sepsis at admission. Because death can preclude the observation of prolonged PICU stay or prolonged mechanical ventilation, we present both full-cohort (non-composite) and composite (death-or-outcome) versions of mechanical ventilation and extended PICU stay to reduce survivor-restriction bias. A cause-specific Cox proportional hazards sensitivity analysis for time to alive PICU discharge is provided in Supplementary Table S1e.

b Adjusted ARDs and NNH were estimated using marginal standardization (g-computation) and were presented only for binary outcomes. NNH was reported only when the ARD 95% CI excluded zero.

c Ninety-five percent CIs for ARDs and NNH were obtained from 2,000 non-parametric bootstrap resamples using the percentile method.

d One patient each had missing data for MODS classification and length of PICU stay and were excluded from the respective analyses.

BH-adjusted *P*-values controlled the false discovery rate across all outcomes in this table using the Benjamini–Hochberg procedure.

For additional secondary outcomes, undernutrition was associated with higher adjusted risks of healthcare-associated infection (35.0% vs. 12.2%; adjusted RR 1.93; 95% CI 1.19–3.14), multiorgan dysfunction syndrome (41.7% vs. 14.4%; adjusted RR 2.55; 95% CI 1.60–4.05), and vasoactive infusion requirement (41.7% vs. 19.6%; adjusted RR 1.51; 95% CI 1.06–2.17); undernourished children also had fewer ventilator-free days to day 28 (adjusted mean difference −3.8 days; 95% CI −6.1 to −1.4). All associations remained statistically significant after adjusting for multiplicity using the Benjamini–Hochberg procedure.

In a cause-specific Cox sensitivity analysis for time to alive PICU discharge, treating death as a censoring event, undernutrition was associated with a lower hazard of discharge (hazard ratio 0.58; 95% CI 0.43–0.78; *P* < 0.001) (Supplementary Table S1e). This finding is consistent with a more prolonged PICU course among undernourished children but should be interpreted cautiously: this model censors children at the time of death, and the lower discharge hazard may reflect both slower clinical recovery and the removal of undernourished children from the discharge risk set due to a higher competing mortality.

### 3.5 Sensitivity and exploratory analyses

#### 3.5.1 Propensity score model

The propensity score model for undernutrition showed good discrimination (AUC 0.803; 95% CI 0.746–0.860). The application of trimmed stabilized inverse probability weighting markedly improved covariate balance across the six included variables, except for age (standardized mean difference 39.7% postweighting) (Supplementary Tables S2a,b). In the propensity score–weighted doubly robust analysis, undernutrition continued to be associated with increased mortality (weighted RR 2.59; 95% CI 1.34–5.03; *P* = 0.005), consistent with the primary analysis (Supplementary Table S3).

#### 3.5.2 Alternative model specifications

In the prespecified sensitivity analyses, alternative model specifications yielded directionally consistent results, although analyses that downweighted or excluded extreme illness severity attenuated the association (Supplementary Table S4 and Supplementary Results). Exploratory analyses suggested directionally consistent associations across illness severity strata, nutritional severity categories, and sepsis strata, with imprecision reflecting the limited number of events (Supplementary Tables S5–S9). The observed mortality substantially exceeded the PIM-3 predicted mortality overall (SMR 4.63), with greater miscalibration among undernourished children (SMR 7.90) than among well-nourished children (SMR 3.65) (Supplementary Table S7).

#### 3.5.3 PICU mortality by undernutrition status stratified by sepsis

The association between undernutrition and PICU mortality was consistent across the sepsis strata. The undernutrition × sepsis interaction was not statistically significant (*P* for interaction = 0.472), providing no evidence that this association differed according to sepsis status (Supplementary Table S8).

#### 3.5.4 PICU mortality by undernutrition status stratified by age

Age-stratified analyses showed directionally consistent but imprecise associations across age groups; neither the linear (*P* = 0.464) nor categorical (*P* = 0.203) interaction test was statistically significant, and the results were materially unchanged after propensity score weighting (Supplementary Table S10 and Supplementary Figure S3), suggesting that the primary association was not meaningfully explained by residual age imbalance.

#### 3.5.5 Net reclassification improvement of undernutrition beyond PIM-3, sepsis, age, and chronic comorbidity

NRI and area under the curve (AUC) analyses were employed to evaluate whether the inclusion of undernutrition enhanced individual-level risk classification beyond the base clinical model. The results indicated that undernutrition did not significantly enhance reclassification or discrimination when incorporated into the base model (Supplementary Tables S11a,b).

#### 3.5.6 E-value analysis for unmeasured confounding

The E-values for the primary and propensity-weighted estimates were 5.00 and 4.62 for the point estimates (Supplementary Table S12), indicating that an unmeasured confounder would require risk ratio associations of approximately 4.6–5.0-fold with both undernutrition and mortality to fully explain their association.

#### 3.5.7 Quantile regression for ventilator-free days at day 28

Median regression confirmed a statistically significant, directionally consistent association between undernutrition and fewer ventilator-free days, supporting the robustness of the primary mean-based estimates (Supplementary Table S13).

## 4 Discussion

### 4.1 Principal findings

In this prospective single-center cohort study, undernutrition at admission was associated with higher PICU mortality after adjusting for PIM-3-predicted mortality, sepsis, age, and chronic comorbidities. Undernutrition was also associated with a more complicated PICU course, including more frequent mechanical ventilation, vasoactive support, prolonged PICU stay, multiorgan dysfunction syndrome, healthcare-associated infection, fewer ventilator-free days, and a lower hazard of alive PICU discharge than that in well-nourished children. However, given the marked overall miscalibration of PIM-3 in this cohort, the adjusted association should be interpreted as hypothesis-generating and potentially subject to residual confounding from inadequately captured illness severity, as discussed in Section 4.3.

### 4.2 Primary outcome

The significant adjusted risk ratio observed aligns with those of recent systematic reviews and meta-analyses, which report mortality hazards typically ranging from 1.5 to 3.8 among undernourished critically ill children (12–15, 26, 27). However, effect sizes differ based on settings, definitions, and methodologies, with some studies identifying weaker or null associations following comprehensive severity adjustment (13, 28). Indian studies have also documented a high prevalence of malnutrition among PICU admissions (18%–40%), although the impact on mortality, after adjusting for illness severity scores, has yielded mixed outcomes (15, 29, 30).

This single-center study offers prospective LMIC-specific data utilizing IAP-specific growth standards for older children, along with doubly robust propensity-weighted sensitivity analyses and formal absolute-risk estimation via g-computation. These methodological features are not consistently present in the studies included in previous meta-analyses (12). These data complement the existing evidence from predominantly high-income settings and provide a basis for future, larger, multicenter investigations.

### 4.3 Sensitivity and exploratory analysis: addressing residual confounding

To assess whether this miscalibration compromises the interpretation of undernutrition as an independently associated factor, we conducted four complementary analyses.

First, miscalibration was identified in both nutritional groups, suggesting that unmeasured severity affects the cohort broadly rather than being specific to the undernutrition–mortality relationship. Second, the association persisted, although at a reduced level, when extreme PIM-3 values were excluded or winsorized. Propensity score weighting yielded a similar estimate (weighted RR: 2.59). Third, the E-value analysis indicated that an unmeasured confounder would need to have risk ratio associations of approximately 4.6–5.0-fold with both undernutrition and mortality to fully explain the association. This threshold is not implausibly high, considering that sepsis, a covariate measured, had an adjusted RR of 3.57. Finally, we assessed whether undernutrition improved individual-level risk classification beyond PIM-3 and other covariates using NRI and found that it did not significantly enhance reclassification or discrimination in this cohort. Given that this analysis relied on apparent non-optimism-corrected predictions in a cohort with 46 deaths, the NRI findings should be interpreted with caution.

Collectively, these four complementary analyses provide moderate reassurance against residual confounding because of inadequately captured illness severity; however, they do not fully address the concern with regard to miscalibration. A future study specifically designed for recalibration may ascertain whether nutritional status contributes additional value in larger, more adequately powered cohorts (15, 16, 31, 32).

We conducted separate evaluations to determine whether sepsis or age, which exhibited significant variation among nutritional groups, contributed to the observed association. The analysis revealed that the relationship between undernutrition and mortality did not significantly vary with sepsis status (P for interaction = 0.472), indicating that sepsis is unlikely to serve as an effect modifier. Nonetheless, residual confounding due to sepsis severity or timing cannot be completely excluded (Section 4.7). Similarly, age-stratified analyses and formal interaction tests performed in both the primary cohort and within a propensity-weighted framework did not provide significant evidence that age modified this association, regardless of whether linear or categorical methods were employed. The overall association remained consistent across the three frameworks. A flexible spline-based test suggested a potential non-linear effect modification; however, given the limited number of deaths relative to the additional parameters required, this finding should be regarded as exploratory rather than confirmatory.

### 4.4 Secondary outcomes analysis

Undernutrition was also associated with several domains of PICU morbidity after adjusting for severity and multiplicity, reinforcing the primary mortality finding. The analyses of mechanical ventilation and prolonged PICU stay employed full-cohort composite endpoints that included death, thereby reducing survivor-restriction bias compared with analyses limited to survivors. These associations align with previous reports indicating longer PICU stays and increased morbidity among undernourished critically ill children (14, 15, 26), as well as meta-analyses linking nutritional deficits to extended hospitalization and increased organ support (12). The elevated incidence of healthcare-associated infections in undernourished children is consistent with previous cohort data (26) and is biologically plausible, given the established immunological and physiological effects of undernutrition, as elaborated in Section 4.5. For ventilator-free days up to day 28, which exhibited a pronounced ceiling effect, median regression confirmed a statistically significant and directionally consistent association with the original mean-based estimate, thereby supporting the robustness of this finding, despite violations of normality.

Healthcare-associated infections and multiorgan dysfunction syndrome may partly lie along the pathway of harm linking undernutrition to mortality, rather than functioning as fully independent endpoints. Immune impairment related to undernutrition may increase susceptibility to infection and organ dysfunction, which may subsequently contribute to mortality. A formal mediation analysis was not conducted, as it would necessitate additional assumptions regarding temporal sequencing and the absence of unmeasured confounding between the mediator and outcome, which our data do not fully support. Instead, they are presented as correlated downstream indicators of a shared underlying process.

### 4.5 Biological mechanisms

Undernutrition impairs immunity, weakens respiratory muscles, and alters lung structure (8, 9, 33). Critical illness accelerates proteolysis and lipolysis, further depleting limited reserves (10, 34–36), and impaired gut barrier integrity may facilitate bacterial translocation (37, 38). Although these mechanisms provide biological plausibility for the observed associations, none were directly measured in this cohort, and mechanistic explanations therefore remain inferential rather than data-derived.

### 4.6 Clinical implications

Although undernutrition remains independently associated with mortality, the lack of significant improvement in the Nutritional Risk Index (NRI) or discrimination suggests limited additional value for individual patient-level prognostic prediction beyond the PIM-3 and existing clinical covariates.

The high prevalence of undernutrition (18%) and its correlation with mortality and various domains of Pediatric Intensive Care Unit (PICU) morbidity indicate that anthropometric screening upon admission may be beneficial for broad clinical risk stratification in high-burden settings. In resource-limited PICU environments, where point-of-care severity scores may inadequately capture nutritional vulnerability, a straightforward, standardized anthropometric assessment at admission, requiring only calibrated scales and a stadiometer or length board, depending on age, could serve as an accessible and low-cost supplement to existing triage processes. Identifying children with z-scores below −2 at admission may assist clinical teams in anticipating the need for more intensive monitoring, nutritional support planning, and resource allocation, even in the absence of validated nutritional risk screening tools in the PICU.

Given that this observational design cannot establish causality, the question whether the systematic implementation of anthropometric screening enhances clinical decision-making or patient outcomes requires prospective evaluation (39–41). The effectiveness of targeted nutritional interventions remains to be demonstrated. The associations observed in this study provide a basis for future research to evaluate whether early targeted nutritional strategies can improve outcomes in critically ill undernourished children (42, 43).

### 4.7 Limitations

This study has several important limitations. First, the substantial overall miscalibration of PIM-3 in this cohort remains a fundamental constraint on causal inference, as discussed in Section 4.3; residual confounding from inadequately captured illness severity cannot be excluded.

Second, as a single-center study conducted in a tertiary Indian PICU with a high prevalence of undernutrition, its generalizability to other Indian settings and diverse LMIC contexts is uncertain. The findings of this study may not extend to high-income country PICUs, where the prevalence, clinical phenotype, and care standards for undernutrition differ substantially.

Third, the modest number of deaths (*n* = 46) limits the precision of estimates; bootstrap validation revealed that the 95% CI for the adjusted risk ratio included the null value of 1.0 in approximately 16% of resamples, indicating meaningful uncertainty in the precise magnitude of the association despite a consistently elevated median estimate.

Fourth, although trimmed stabilized inverse probability weighting substantially improved covariate balance, age retained the largest residual standardized mean difference after weighting (39.7%). Given the substantial age disparity between the two groups (median 1.50 vs. 5.08 years), incomplete adjustment for age may persist, and the propensity-weighted estimate (aRR 2.59) should therefore be interpreted with caution.

Fifth, approximately 11% of screened admissions were excluded, mainly because reliable anthropometric measurements could not be obtained or because of early transfer or discharge against medical advice. Among the excluded children with baseline and outcome data available (*n* = 28, comprising 24 with anthropometry failure and 4 with gross edema), median PIM-3 predicted mortality (1.5%) and observed mortality (10.7%) were comparable to the analytic cohort overall (PIM-3, 1.3%; mortality, 13.9%), arguing against the systematic exclusion of the most severely ill children in this recoverable subset. However, nutritional status could not be determined for any excluded child; therefore, selection bias affecting the undernutrition-mortality relationship cannot be excluded. Outcome data were unavailable for the 10 children transferred within 24 h and the 5 who left against medical advice. Anthropometric measurements were obtained during acute illness when fluid resuscitation and hemodynamic instability may have influenced weight. Nutritional status was assessed at a single time point, limiting the ability to distinguish chronic undernutrition from acute illness–related decline. The possibility of reverse causation, whereby severe illness causes both nutritional deterioration and subsequent mortality, cannot be ruled out.

Sixth, although sepsis was included in multivariable adjustment and did not significantly modify the undernutrition–mortality association (Section 4.3), residual confounding remains possible because sepsis severity, infection source, organ dysfunction burden, and timing of presentation were not fully characterized. More broadly, despite comprehensive adjustment and sensitivity analyses, residual confounding by unmeasured factors, including baseline functional status, socioeconomic status, pre-PICU care quality, chronic nutritional trajectories, micronutrient status, and pre-PICU or in-PICU nutritional interventions, cannot be excluded and may bias the observed association in either direction.

Finally, this study evaluated only in-PICU and short-term outcomes and did not capture postdischarge mortality, rehospitalization, neurodevelopmental outcomes, or functional recovery. We conducted multiple secondary outcome analyses (*n* = 8, adjusted for multiplicity using the Benjamini–Hochberg procedure) and exploratory analyses (*n* = 8, not adjusted for multiple comparisons); some positive exploratory findings may reflect Type I error.

### 4.8 Future directions

Future research should prioritize multicenter prospective cohorts in diverse LMIC settings with standardized anthropometric assessment, longitudinal nutritional measurement, and postdischarge follow-up. Pragmatic cluster-randomized trials are needed to evaluate whether systematic anthropometric screening and targeted nutritional interventions can reduce mortality and morbidity in undernourished critically ill children (43, 44). Methodological work to recalibrate severity-of-illness scores for LMIC populations and evaluate whether incorporating anthropometric measures improves discrimination and calibration would enhance prognostic model performance.

## 5 Conclusions

In this single-center prospective cohort study, undernutrition was associated with higher PICU mortality and multiple adverse short-term outcomes after adjusting for PIM-3-predicted mortality, sepsis, age, and chronic comorbidities. These observational findings suggest that anthropometric screening at PICU admission may have prognostic value in high-undernutrition settings and that existing severity-of-illness models may warrant local validation in such populations. However, causal inference is limited by residual confounding, including unmeasured illness severity potentially contributed to by PIM-3 miscalibration, reverse causation, and the single-center observational design. Larger multicenter studies are needed to confirm these associations across diverse LMIC settings, and pragmatic interventional trials need to be conducted to determine whether early, targeted nutritional interventions can reduce mortality and morbidity in undernourished critically ill children. Until such evidence becomes available, these findings support the routine consideration of standardized anthropometric assessment at PICU admission in settings with a high-undernutrition burden—a simple, feasible, and low-cost practice that may meaningfully inform clinical risk stratification and resource planning for the most vulnerable children.

## Data Availability

The raw data supporting the conclusions of this article will be made available by the authors, without undue reservation.
