# Supplementary material for: Association between admission undernutrition and mortality rates and adverse outcomes in a pediatric intensive care unit in India: a prospective cohort study
Source: Front Pediatr. 2026 Aug 17;14:1880294. doi: 10.3389/fped.2026.1880294 (PMC13526918; doi:10.3389/fped.2026.1880294)
Supplement: Supplementary file 1 [file Supplementaryfile1.docx]

Supplementary Material

SUPPLEMENTARY DIGITAL CONTENT

**Association Between Admission Undernutrition and Mortality Rates and Adverse Outcomes in a Pediatric Intensive Care Unit in India: A Prospective Cohort Study**

Vijay Raghunath Kalrao, MD; Aastha Pramod, MD

TABLE OF CONTENTS

| **Content Type** | **Description** | **Page Number** |
| --- | --- | --- |
| **Supplementary Methods** |  | 7 |
|  | Extended Setting Description | 7 |
|  | Detailed Anthropometric Protocol | 7 |
|  | Statistical Methods & Sample Size Estimation | 8 |
|  | Primary Analysis Specification | 8-9 |
|  | Model & Bootstrap Validation Methods | 9-10 |
|  | Propensity Score Methodology | 10-11 |
|  | Sensitivity analysis with Alternative Model Specifications | 11 |
|  | Exploratory Analysis Specifications | 11 |
|  | Mortality with Undernutrition Status Stratified by Illness Severity | 11 |
|  | Dose-Response Across Undernutrition Severity | 12 |
|  | Standardized Mortality Ratios | 12 |
|  | PICU Mortality by Undernutrition Status Stratified by Sepsis | 12 |
|  | Excluding Trauma/Toxicology Admissions | 12 |
|  | Stratified Mortality, and Age-Interaction Analyses for the Undernutrition-Mortality Association | 13 |
|  | Net Reclassification Improvement of undernutrition beyond PIM-3, sepsis, age, and chronic comorbidity | 13 |
|  | E-Value Analysis for Unmeasured Confounding | 13 |
|  | Ventilator-Free Days to Day 28: Distribution and Quantile Regression Sensitivity Analysis | 13 |
| **Supplementary Results** |  | 14 |
|  | Chronic Comorbidity Categories by Organ System according to Nutritional Status | 14 |
|  | Anthropometric deficits at PICU admission by age group | 14 |
|  | Anthropometric summary of the full cohort according to undernutrition status, median (IQR) | 14 |
|  | Bootstrap Validation for Adjusted Relative Risk | 14 |
|  | Model Calibration and Predicted Risk Distribution | 15 |
|  | Sensitivity Analysis for Time to Alive PICU Discharge | 15 |
|  | Propensity Score Methodology, Weighting, Discrimination and Doubly Robust Analysis | 15 |
|  | Sensitivity Analysis with Alternative Model Specifications | 15 |
|  | Multicollinearity Assessment | 16 |
|  | Mortality by Undernutrition Status Stratified by PIM-3 Tertile | 16 |
|  | Dose-Response Analysis Across Undernutrition Severity | 16 |
|  | Standardized Mortality Ratios | 16 |
|  | PICU Mortality by Undernutrition Status Stratified by Sepsis | 17 |
|  | Excluding Trauma/Toxicology Admissions | 17 |
|  | Stratified Mortality, and Age-Interaction Analyses for the Undernutrition-Mortality Association | 17 |
|  | Net Reclassification Improvement of undernutrition beyond PIM-3, sepsis, age, and chronic comorbidity | 18 |
|  | E-Value Analysis for Unmeasured Confounding | 18 |
|  | Ventilator-Free Days to Day 28: Distribution and Quantile Regression Sensitivity Analysis | 18 |
| **Supplementary**  **Figure 1** | Predicted Risk Distribution | 19 |
| **Supplementary Tables** |  | 20 |
| **Supplementary**  **Table S1a** | Chronic Comorbidity Categories by Organ System according to Nutritional Status | 20 |
| **Supplementary**  **Table S1b** | Anthropometric deficits at PICU admission by age group | 21 |
| **Supplementary**  **Table S1c** | Anthropometric summary of the full cohort according to undernutrition status, median (IQR) | 22 |
| **Supplementary**  **Table S1d** | Bootstrap Validation for Adjusted Relative Risk | 23 |
| **Supplementary**  **Table S1e** | Cause-Specific Cox Sensitivity Analysis for Time to Alive PICU Discharge | 24 |
| **Supplementary**  **Table S2a** | Absolute Standardized Mean Differences (PS Balance) | 25 |
| **Supplementary**  **Table S2b** | Propensity Score & IPTW Diagnostics | 26-27 |
| **Supplementary**  **Table S3** | Propensity Score–Weighted, Doubly Robust Analysis | 28 |
| **Supplementary Table S4** | Sensitivity Analyses with Alternative Model Specifications | 29-30 |
| **Supplementary Table S5** | Mortality Stratified by PIM-3 Tertile | 31 |
| **Supplementary Table S6** | Dose-Response Analysis Across Undernutrition Severity | 32 |
| **Supplementary Table S7** | Standardized Mortality Ratios Overall and by Status | 33 |
| **Supplementary Table S8** | PICU Mortality Stratified by Sepsis at Admission | 34 |
| **Supplementary Table S9** | Sensitivity Analysis: Excluding Trauma/Toxicology | 35 |
| **Supplementary Table S10** | Age Distribution, Stratified Mortality, and Age-Interaction Analyses for the Undernutrition-Mortality Association | 36-37 |
| **Supplementary Table S11a** | Net Reclassification Improvement of Undernutrition Beyond PIM-3, Sepsis, Age, and Chronic Comorbidity | 38 |
| **Supplementary Table S11b** | Discrimination Comparison Between Base and Extended Models | 39 |
| **Supplementary Table S12** | E-Value Analysis for Unmeasured Confounding | 40 |
| **Supplementary Table S13** | Ventilator-Free Days to Day 28: Distribution and Quantile Regression Sensitivity Analysis | 41 |
| **Supplementary Figures** |  | 42 |
| **Supplementary Figure 1** | Distribution of Predicted PICU Mortality Risk | 42 |
| **Supplementary Figure 2.** | Calibration of the prespecified PICU mortality prediction model (ridge-penalized logistic regression). | 43 |
| **Supplementary Figure 3** | Adjusted mortality risk by age and nutritional status | 44 |
| **STROBE CHECKLIST** |  | 45-49 |
| **REFERENCES** |  | 49 |

**SUPPLEMENTARY METHODS**

**Extended Setting Description**

The study was conducted in a 16-bed mixed medical-surgical PICU that admits approximately 350–400 patients annually. The unit population consists of 80% medical and 20% surgical/trauma/toxicology cases, primarily from lower-middle-income urban and rural backgrounds. Care is led by full-time pediatric intensivists, with nurse-to-patient ratios of 1:2 for non-ventilated patients and 1:1 for ventilated patients. The center does not perform cardiac surgery or solid organ transplantation.

**Detailed Anthropometric Protocol**

Anthropometric measurements obtained within 24 hours of PICU admission were used to classify nutritional status using age-appropriate reference standards. When measurement was not feasible at admission due to hemodynamic instability, the most recent reliable measurement from the preceding seven days was recorded. For children younger than 5 years, the World Health Organization standards were used, and undernutrition was defined as weight-for-length/height z-score. For children aged 5 to 18 years, the Indian Academy of Pediatrics references were used, and undernutrition was defined using the BMI-for-age z-score. Underweight and stunting were defined as z-scores < −2SD, and severe deficits were defined as z-scores < −3SD.

Weight was measured using a digital scale calibrated to the nearest 10 g (for infants) or 100 g (for older children). Recumbent length was measured for children <2 years of age using an infantometer, and standing height for children ≥2 years of age using a stadiometer, both to the nearest 0.1 cm. For sensitivity analyses, nutritional status was further classified into five ordered categories: severe undernutrition (z < −3SD), moderate undernutrition (−3SD ≤ z < −2SD), normal nutrition (−2SD ≤ z ≤ +1SD), overweight (+1SD < z ≤ +2SD), and obesity (z > +2SD) (1,2).

**Exclusion Criteria:** Children with gross edema (pitting edema ≥2 body regions), severe skeletal deformities preventing accurate measurement, organomegaly, or ascites that would distort anthropometric measurements were excluded to maintain the validity of the nutritional classification.

**Statistical Methods: Complete Specifications**

***Sample Size Calculation*:** We estimated that 185 participants would provide 90% power (two-sided alpha 0.05) to detect the hypothesized mortality difference, assuming 19% undernutrition prevalence, 16% mortality in well-nourished children, and 45% mortality in undernourished children (absolute risk difference 29 percentage points, corresponding to risk ratio approximately 2.8). These assumptions were based on the National Family Health Survey data on undernutrition prevalence, institutional historical mortality data, and prior meta-analyses reporting hazard ratios of 1.5–3.8 for mortality associated with undernutrition (3,4). We prospectively enrolled all consecutive eligible admissions over 22 months, yielding 331 participants who substantially exceeded the required sample size. The actual observed mortality difference (19.7 percentage points) was smaller than assumed, but the larger-than-planned sample size compensated for this, providing adequate power for the observed difference.

***Missing Data*:** We performed a complete case analysis for each outcome. Missingness was minimal (<1% for any variable): one patient missing MODS classification (end-of-life care de-escalation precluded complete organ dysfunction scoring) and one missing extended PICU stay (inter-facility transfer on day 3). No imputations were performed on the data.

**Primary Analysis:** We used modified Poisson regression with a log link and robust standard errors (Huber-White sandwich estimator) to estimate adjusted risk ratios, as this approach directly estimates relative risks rather than odds ratios and is more appropriate for outcomes with a prevalence >10%. The model specification was log(P[mortality]) = β₀ + β₁(undernutrition) + β₂(age) + β₃(PIM-3) + β₄(sepsis) + β₅(chronic condition). Robust standard errors account for the variance misspecification that is inherent in the use of Poisson regression for binary outcomes.

Covariates were selected a priori based on clinical relevance and prior literature. Covariates included in the primary multivariable model were selected a priori during protocol development based on clinical knowledge and prior literature on covariates for pediatric intensive care outcomes (5-8), without reference to their association with outcomes in this dataset: age (continuous, per year), PIM-3 predicted mortality (continuous, per 1-SD increase), sepsis at admission (binary), and chronic comorbidities (binary). The goal was to create a parsimonious model that was adjusted for key confounders without over-adjusting for potential mediators or colliders.

***Assessment of Multicollinearity*:** We evaluated multicollinearity among predictors using Pearson’s correlation coefficients for continuous variables (age and PIM-3 predicted mortality) and variance inflation factors (VIF) from the primary multivariable logistic regression model. The correlation between age and PIM-3 was negligible (r=0.02), indicating independence between these continuous covariates. All VIF values were below 1.25 (undernutrition 1.22, age 1.14, PIM-3 predicted mortality 1.07, sepsis at admission 1.11, chronic/comorbid condition 1.17), well below the conventional thresholds for concern (VIF <5 or <10), confirming the absence of meaningful multicollinearity that could affect coefficient stability or interpretation.

***Absolute Risk Differences****:* For binary exposures, adjusted absolute risk differences (ARDs) and numbers needed to harm (NNH) were estimated using marginal standardization (g-computation). We fitted a logistic regression model with the same predictors, generated two predicted probabilities for each participant (assuming undernourished vs. well-nourished while holding the covariates constant), and calculated the ARD as the mean individual-level difference. NNH = 1/|ARD|. Bootstrap 95% CIs (1,000 resamples, percentile method) were obtained for ARDs and NNHs. (**Table 2**)

**Model Validation**

With 46 events and five predictors, the event-per-variable ratio was 9.2, supporting the model fitting despite the modest event count. Model discrimination and calibration were assessed using a prespecified logistic regression model that included undernutrition, age, PIM-3 predicted mortality, chronic comorbid conditions, and sepsis at admission. This logistic model was used only for validation; primary inference regarding risk ratios was based on modified Poisson regression and marginal standardization of the absolute risk differences.

Apparent performance was calculated in the full cohort using a standard (unpenalized) logistic model, with discrimination summarized by the area under the receiver operating characteristic curve (AUC). Calibration was assessed using calibration-in-the-large and calibration slope, supplemented by decile-based calibration plots (**Figures 2, main manuscript; Supplementary Figures 1 and 2 )**). The calibration slope quantifies whether the predictions are systematically too extreme or conservative, with a slope of 1 indicating ideal calibration and values of < 1 indicating overfitting.

Internal validation used 1,000 bootstrap resampling iterations. In each resample, the model was refitted using ridge-penalized logistic regression (L2 penalty, lambda selected by cross-validation) to mitigate quasi-complete separation, which occurs when sparse covariate patterns yield extremely predicted probabilities. In each bootstrap replicate, the performance was evaluated using both the bootstrap sample and the original dataset to estimate optimism. The optimism-corrected AUC and calibration slope were obtained by subtracting the mean optimism from apparent estimates. Decile-based calibration plots for both unpenalized and ridge-penalized models are provided in **Figures 2 (main manuscript) and Supplementary Figure 1 and 2**, along with the distribution of the predicted probabilities.

**Bootstrap Validation of Primary Effect Estimate**

We evaluated the stability of the adjusted undernutrition-mortality association using non-parametric bootstrap resampling. The adjusted risk ratio was estimated using a prespecified modified Poisson regression model with robust standard errors. We generated 1,000 resamples with replacement and refitted the model for each of the resamples. The bootstrap distribution was summarized using the mean and median risk ratios, with uncertainty quantified using the percentile and bias-corrected and accelerated 95% confidence intervals. As a descriptive measure of robustness, we report the proportion of bootstrap refits in which the resample-specific Wald 95% confidence interval excluded 1.0 (**Supplementary Table** **S1d, and Supplementary Figures** **1 and 2**)

**Sensitivity and Exploratory Subgroup Analyses**

We performed multiple exploratory subgroup and sensitivity analyses. These analyses were hypothesis-generating and were interpreted based on effect sizes and confidence intervals rather than formal control of the family wise error rate. Therefore, the p-values from the exploratory analyses were not adjusted for multiple comparisons.

***Sensitivity Analyses (prespecified)***

1. Propensity score weighting: Detailed methods are provided in the following section.
2. Sensitivity analysis with alternative model specifications
   i. Alternative PIM-3 specifications: Modeled as tertiles rather than continuous.
   ii. Extreme severity: Excluded PIM-3 ≥97.5th percentile; separately, winsorized at 95th percentile
   iii. Exclusion of obese children: Removed z>+2 from reference group.

**Exploratory Analyses (Not Adjusted for Multiple Testing)**

1. Effect modification by severity: Mortality by undernutrition within PIM-3 tertiles; interaction test using Firth penalized logistic regression.
2. Dose-response: Three-category nutritional classification with test for linear trend.
3. Standardized mortality ratios: Observed/expected deaths using PIM-3 predictions.
4. Sepsis stratification: Association within sepsis strata; interaction test.
5. Trauma/toxicology exclusion: Repeated primary analysis excluding these admissions.

Exploratory analyses were hypothesis-generating; P-values were nominal and may reflect Type I error.

**Propensity Score Methodology, Weighting, Model Diagnostics and Doubly Robust Sensitivity Analysis**

We conducted a prespecified sensitivity analysis using propensity score methods to further address the confounding factors in the association between undernutrition and PICU mortality. The propensity score for undernutrition (undernourished vs. well-nourished) was estimated using multivariable logistic regression, including age, female sex, maternal education dichotomized as graduate/postgraduate vs. other, PIM-3 predicted probability of death, chronic/comorbid condition, and sepsis at admission. Stabilized inverse probability of treatment weights were calculated as the marginal prevalence of the observed exposure divided by each individual’s predicted probability of the observed exposure. To limit the influence of extreme weights, stabilized weights were trimmed at the 1st and 99th percentiles and rescaled to have a mean of 1; a prespecified cap of 20 was applied if necessary. Covariate balance before and after weighting was assessed using absolute standardized mean differences **(Supplementary Table S2a**), and propensity score model discrimination was summarized using the area under the receiver operating characteristic curve (AUROC) (**Supplementary Table** **S2b**). The association between undernutrition and PICU mortality was estimated using a weighted modified Poisson regression model with a log link and robust standard errors. For a doubly robust estimate, the weighted outcome model was adjusted for age, PIM-3 predicted probability of death, chronic/comorbid conditions, and sepsis at admission (**Supplementary Table** **S3).**

**Sensitivity Analysis with Alternative Model Specifications**

Prespecified sensitivity analyses were used to evaluate the robustness of the association between undernutrition and PICU mortality in alternative model specifications and the potential influence of extreme illness severity. Relative risks were estimated using modified Poisson regression with a log link and robust standard errors. The primary adjusted model included undernutrition (undernourished vs. well-nourished) and covariates such as age in years, PIM-3 predicted probability of death entered per 1-SD increase, chronic/comorbid conditions, and sepsis at admission. We refitted the primary modified Poisson model using an alternative functional form for illness severity by entering PIM-3 predicted mortality tertiles (T1–T3) rather than continuous PIM-3 per 1-SD increase, with all other covariates remaining unchanged. We also fitted a parsimonious model that included undernutrition, PIM-3 tertiles (T1–T3), and sepsis at admission. To assess sensitivity to extreme values of illness severity, we repeated the primary model after excluding children with PIM-3 at or above the 97.5th percentile (PIM-3 ≥10.25and separately, after winsorizing PIM-3 by capping values above the 95th percentile (PIM-3 capped at 8.55) while retaining all children. We additionally repeated the primary model after excluding overweight/obese children defined as BMI-for-age or weight-for-height z-score >+2 (**Supplementary Table** **S4).**

**Exploratory Analysis Specifications**

***Mortality with undernutrition status stratified by Illness Severity***

For exploratory analyses examining potential effect modification by illness severity, the PIM-3 predicted mortality at admission was categorized into tertiles based on the observed distribution in the analysis cohort. Tertile boundaries were defined using the 33rd and 67th percentiles of the PIM-3 distribution: Tertile 1 (T1, lowest severity): PIM-3 ≤0.2% (n=121); Tertile 2 (T2, moderate severity): PIM-3 0.3–2.9% (n=107); and Tertile 3 (T3, highest severity): PIM-3 >2.9% (n=103) **(Supplementary Table S5).**

Within each tertile, we calculated the proportion of deaths among undernourished and well-nourished children and estimated relative risks (RRs) and 95% confidence intervals using log-transformed Wald methods, when estimable. When either group had zero deaths in a given tertile, RRs and CIs were not calculated and are reported as "Not estimable." To explore the potential effect modification by illness severity, we fitted a logistic regression model including a nutritional status × PIM-3 tertile interaction term and obtained a likelihood ratio test P-value. Firth penalization was used to address sparse events and separations in the data. This interaction analysis was exploratory, given the limited number of deaths.

***Dose-Response Across Undernutrition Severity***

To examine the dose-response relationship between undernutrition severity and PICU mortality, we categorized nutritional status using BMI-for-age or weight-for-height/length z-scores (WHO/IAP standards) as normal (z ≥ 2SD), moderate undernutrition (-3SD ≤ z <–2SD), and severe undernutrition (z <–3SD). For each category, we calculated deaths, totals, and risks and estimated crude RRs with 95% CIs using Poisson regression with a log link and robust standard errors. The adjusted models included nutritional category, PIM-3 tertiles, and sepsis at admission. A test for linear trends across ordered nutritional categories was performed by modeling the category as an ordinal variable in the adjusted Poisson model and reporting the corresponding P values ( **Table S6).**

***Standardized Mortality Ratios***

Standardized mortality ratios (SMRs) were calculated overall and by nutritional status using PIM-3 -based expected mortality. For each child, the expected probability of death was obtained from the recorded PIM-3 predicted mortality value (recorded as a percentage) and converted to a probability by dividing it by 100. The expected deaths in each group were calculated as the sum of the individual expected probabilities. The SMR was defined as the ratio of observed to expected deaths, with values >1.0 indicating higher-than-expected mortality and values <1.0 indicating lower-than-expected mortality. Ninety-five percent CIs for SMRs were calculated using an exact Poisson (chi-square) method for the observed number of deaths. These analyses were exploratory assessments of the PIM-3 calibration overall and potential differential calibration by nutritional status (**Supplementary Table S7).**

***PICU Mortality by Undernutrition Status Stratified by Sepsis***

To evaluate whether sepsis at admission modified the association between undernutrition and PICU mortality, we conducted a stratified analysis based on the sepsis status at admission. Within each stratum, crude risk ratios (RRs) and 95% CIs were calculated using 2×2 tables. Adjusted RRs were estimated separately within each stratum using modified Poisson regression with a log link and robust standard errors, adjusting for age (years), PIM-3 predicted mortality, and chronic comorbid conditions. Effect modification was assessed in the full cohort by adding an undernutrition × sepsis interaction term to the adjusted modified Poisson model; the reported P value for interaction corresponds to this term (**Supplementary Table** **S8)**

***Excluding Trauma/Toxicology Admissions***

As a sensitivity analysis, we repeated the primary analysis of the association between undernutrition and PICU mortality after excluding children admitted with trauma or toxic diagnoses. Undernutrition (undernourished vs. well-nourished) was the exposure, and PICU mortality was the outcome variable. Crude risk ratios (RRs) were calculated using 2×2 tables. Adjusted RRs were estimated using modified Poisson regression with a log link and robust standard errors, adjusting for age (years), PIM-3 predicted mortality at admission, chronic/comorbid conditions, and sepsis at admission. This sensitivity analysis evaluated the robustness of the primary findings after excluding trauma and toxicology admissions **(Supplementary Table S9)**.

***PICU mortality by undernutrition status stratified by age***

Because undernourished and well-nourished children differed substantially in age and residual age imbalance persisted after propensity score weighting (**Supplementary Table S2a**), we conducted additional age-focused analyses. We stratified the cohort into three clinically relevant age categories (<1, 1–5, and >5 years; children aged exactly 5 years were classified in the 1–5 year category) and estimated the undernutrition-mortality association within each stratum using modified Poisson regression adjusted for PIM-3 predicted mortality, sepsis, and chronic comorbid conditions. We then formally tested for effect modification by age using three nested approaches: a continuous linear undernutrition × age interaction term, a categorical undernutrition × age-category joint Wald test, and a more flexible undernutrition × restricted cubic spline age joint Wald test. All analyses were repeated within the trimmed, stabilized inverse probability of treatment weighting framework described earlier. These age-interaction analyses were exploratory and were not adjusted for multiple comparisons (**Supplementary Table S10).**

***Net Reclassification Improvement of undernutrition beyond PIM-3, sepsis, age, and chronic comorbidity***

To assess whether undernutrition added prognostic information beyond illness severity and clinical covariates, we compared a base logistic model (PIM-3 predicted mortality, sepsis at admission, age, and chronic comorbid conditions) with an extended model that included undernutrition status. We calculated the continuous net reclassification improvement (NRI) using apparent (within-sample) predicted probabilities from both models, with 95% confidence intervals from 1,000 bootstrap resamples. We also compared the discrimination (AUROC) and Brier scores between the models. Because this analysis used apparent, non-optimism-corrected predictions from a model with known sensitivity to sparse covariate patterns (Figures 2, main manuscript, and Supplementary Figures 1 and 2) and a modest number of deaths (n=46),the results are presented as exploratory only ((**Supplementary Tables S11a and S11b)**.

***E-value Analysis for Unmeasured Confounding***

To quantify the sensitivity of the undernutrition-mortality association to unmeasured confounding, we calculated E-values using the method described by VanderWeele and Ding (10). The E-value represents the minimum risk ratio that an unmeasured confounder would need with both undernutrition and mortality, conditional on measured covariates, to fully explain the observed association. E-values were calculated for both the point estimate and the lower bound of the 95% confidence interval using the primary adjusted modified Poisson estimate and the propensity score-weighted doubly robust estimate (**Supplementary Table S12**).

***Ventilator-Free Days to Day 28: Distribution and Quantile Regression Sensitivity Analysis***

Because ventilator-free days to day 28 (VFD-28) showed a marked ceiling effect and non-normal distribution, we additionally performed median regression (quantile regression, τ=0.50) as a sensitivity analysis, adjusting for the same covariates as the primary linear regression model. Ninety-five percent confidence intervals were obtained using 1,000 bootstrap resamples (**Supplementary Table S13**).

**Software:**

Statistical analyses were conducted using *R* version 4.4.3 with the packages dplyr (1.1.4), readxl (1.4.3), sandwich (3.1-0), boot (1.3-30), pROC (1.18.5), glmnet (4.1-8), WeightIt (1.2.0), cobalt (4.5.5), logistf (1.26.0), car (3.1-2), and survival (3.8-6).

**SUPPLEMENTARY RESULTS**

**Chronic comorbidity categories by organ system according to nutritional status**

**Supplementary Table S1a** Chronic comorbidity categories by organ system according to the nutritional status. Hematology-oncology was the most frequent chronic comorbidity category (28 of 90 [31.1%]), followed by other comorbidities (27 of 90 [30.0%]) and neurologic comorbidities (15 of 90 [16.7%] patients). Renal comorbidity was uncommon (4 of 90 [4.4%]) and occurred only in well-nourished children. The distribution of comorbidity categories did not differ significantly according to nutritional status (P = .32).

**Anthropometric deficits at PICU admission by age group and Anthropometric summary of the full cohort according to undernutrition status, median (IQR)**

**Supplementary Tables S1b and S1c** summarize the age-stratified anthropometric deficits at PICU admission and the admission anthropometric characteristics according to undernutrition status**.** Of the 331 children, 181 were younger than 5 years and 150 were aged 5–18 years. Anthropometric deficits, including underweight, stunting, and composite undernutrition, were more prevalent in younger children. Overall, 60 children (18.1%) met the criteria for underweight, 76 (23.0%) for stunting, and 60 (18.1%) for composite undernutrition; severe undernutrition was present in 29 children (8.8%). Undernourished children had lower admission weights, shorter lengths/heights, and lower composite anthropometric z-scores than well-nourished children did.

**Bootstrap Internal Validation**

Among the 331 children, 46 died (EPV: 9.2). The adjusted RR for PICU mortality comparing undernourished and well-nourished children was 2.78 (Wald 95% CI 1.58–4.91). In 1,000 bootstrap resamples, the RR distribution had a median of 2.51 and mean of 2.64; the 95% CI was 1.20–4.83 and the BCa 95% CI was 1.48–6.10, which showed adjustment for distribution skewness. In 16.3% of the resamples, the CIs crossed unity, reflecting limited precision for 46 events. However, 92.3% of bootstrap estimates exceeded 1.5, and 68.2% exceeded 2.0, supporting the robustness of directional findings despite uncertainty about the exact magnitude (**Supplementary Table S1d, Supplementary Figure** **1 and 2 ; Figure 2 in the main manuscript**). Overall, the bootstrap distribution was centered close to the original estimate, supporting the stability of the estimated association with sampling variations. The bootstrap CIs remained largely above 1.0 and were similar in magnitude to the standard model-based CI, suggesting that the primary inference was not driven by a small number of influential observations. A wider BCa interval indicates some skew and/or influence of resamples with fewer events, consistent with the uncertainty expected at this event rate (**Supplementary Table** **S1d).**

**Model Calibration and Predicted Risk Distribution (Supplementary Figures 1)**

The distribution of the predicted mortality probabilities **(Supplementary Figure 1**) showed that the extreme predicted values were rare. Under the unpenalized model, the predicted risks ranged from 0.001 to 1.000, with the 99th percentile at 0.984, and only two observations exceeded 0.999. Under the ridge model, the predicted risks ranged from 0.009 to 1.000, with one observation exceeding 0.999 and a slightly less-extreme upper tail.

Together, the calibration plots and risk distributions support that the model's discrimination and calibration were not driven by a small number of near-deterministic predictions and that the validation results were robust to the ridge penalization.

**Cause-Specific Cox Sensitivity Analysis for Time to Alive PICU Discharge**

The cause-specific Cox model estimated the instantaneous hazard of alive PICU discharge among children who were still alive and not yet discharged at each time point; a lower hazard ratio indicated slower recovery and longer PICU stay.

**Propensity Score Weighting, Discrimination and Doubly Robust Analysis**

The propensity score model showed good discrimination for undernutrition (AUROC 0.803, 95% CI, 0.746–0.860). After applying the trimmed stabilized inverse probability of treatment weights, the covariate balance improved substantially for all variables. The maximum absolute standardized mean difference decreased from 82.1% to 39.7% (for age), and the mean absolute standardized mean difference across all covariates decreased from 37.3% to 13.3%. Age remained the variable with the largest residual imbalance after weighting. which was substantial but had improved from the baseline. Eight of the 331 observations (2.42%) were affected by trimming, and no weights were required to cap at 20. The effective sample size (ESS) after weighting was 298.7 (90.2% of the original sample), indicating a moderate loss of statistical information (**Supplementary Table** **S2a and S2b).** In the propensity score–weighted doubly robust analysis, undernutrition remained associated with higher PICU mortality (RR 2.59, 95% CI 1.34–5.03; P = 0.005), consistent with the primary analysis (**Supplementary Table S3**).

**Sensitivity Analysis with Alternative Model Specifications**

Across alternative specifications, undernutrition was associated with higher PICU mortality rates. When illness severity was modeled using PIM-3 tertiles rather than as a continuous per-SD term, the adjusted RR was 2.28 (95% CI, 1.36–3.84), and in the parsimonious model including undernutrition, PIM-3 tertiles, and sepsis, the adjusted RR was 1.93 (95% CI, 1.23–3.03). Approaches targeting extreme illness severity attenuated this association but did not change its directionality. Excluding children with PIM-3 ≥ 10.25 (97.5th percentile) yielded an adjusted RR of 1.89 (95% CI, 1.08–3.31), and winsorizing PIM-3 at 8.55 (95th percentile) yielded an adjusted RR of 1.52 (95% CI, 1.02–2.34). After excluding overweight/obese children, the adjusted RR was 2.72 (95% CI: 1.54–4.78). Collectively, these analyses support the robustness of the primary association, with some attenuation when extreme PIM-3 values were downweighted or excluded (**Supplementary Table** **S4).**

**Multicollinearity Assessment:** Assessment of multicollinearity confirmed model stability. Age and PIM-3 predicted mortality showed negligible correlation (r=0.02). All variance inflation factors were below 1.25 (undernutrition 1.22, age 1.14, PIM-3 1.07, sepsis 1.11, and chronic condition, 1.17), well below the thresholds indicating problematic collinearity. These findings support the notion that the covariates were appropriately independent and that the coefficient estimates remained stable.

**Exploratory Analysis**

***Mortality by Undernutrition Status Stratified by PIM-3 Tertile***

In the highest illness severity stratum (T3), mortality was 18/26 (69.2%) among undernourished children and 26/77 (33.8%) among well-nourished children, corresponding to an RR of 2.05 (95% CI 1.37–3.07). In T1, there were no deaths among the undernourished children (0/19) and two deaths among the well-nourished children (2/102 children). In T2, there were no deaths in either group (0/15 vs. 0/92); therefore, stratum-specific RRs were not estimable in these tertiles. The interaction test from the Firth penalized logistic regression did not provide evidence of heterogeneity by tertile (P for interaction = .889). Given the sparse events in the lower tertiles, these stratified analyses should be interpreted as exploratory (**Supplementary Table** **S5).**

Because PIM-3 reflects acute physiologic derangement at admission and may partially lie on the pathway between chronic undernutrition and mortality risk, stratified estimates by PIM-3 tertile may reflect both effect modification and severity-related mediation

***Dose-Response Analysis Across Undernutrition Severity***

In exploratory analyses treating nutritional status as a three-level variable (normal, moderate undernutrition, and severe undernutrition), both moderate and severe undernutrition were associated with higher PICU mortality than normal nutritional status (**Supplementary Table S6**). After adjusting for PIM-3 tertiles and sepsis at admission, the adjusted RR was 1.75 (95% CI, 1.12–2.74; p = .014) for moderate undernutrition and 1.51 (95% CI, 0.96 –2.39; p = .076) for severe undernutrition, compared with normal nutrition. The test for linear trend across the three ordered categories was statistically significant (p for trend = .023), supporting an overall dose-response relationship; small departures from strict monotonicity likely reflect the limited number of deaths in the moderate and severe groups (**Supplementary Table** **S6).**

***Standardized Mortality Ratios***

Among children with complete PIM-3 and outcome data, the observed PICU mortality exceeded the PIM-3 -expected mortality. There were 46 observed deaths versus 9.94 expected deaths, corresponding to an SMR of 4.63 (95% CI 3.39–6.17) (**Supplementary Table S7**). When stratified by nutritional status, SMRs were elevated in both groups but were higher among undernourished children. Well-nourished children had 28 observed versus 7.66 expected deaths (SMR, 3.65; 95% CI, 2.43–5.28), whereas undernourished children had 18 observed versus 2.28 expected deaths (SMR, 7.90; 95% CI, 4.68–12.49).

Differential underprediction by nutritional status indicates that PIM-3 miscalibration is more severe among undernourished children, complicating the interpretation of undernutrition as being independently associated with mortality and suggesting that PIM-3 may inadequately capture the severity dimensions relevant to this population. However, these SMR analyses are exploratory and do not distinguish miscalibration related to nutritional status from other sources of case-mix and setting differences. Further evaluation in external cohorts and potential recalibration or model updating may be warranted before using the PIM-3 for benchmarking across nutritional strata in similar settings (**Supplementary Table S7).**

***PICU mortality by undernutrition status stratified by sepsis***

Among children without sepsis at admission (n=165), mortality was 2/14 (14.3%) in undernourished children and 9/151 (6.0%) in well-nourished children, corresponding to a crude RR of 2.40 (95% CI, 0.57–10.03) and an adjusted RR of 4.51 (95% CI, 1.37–14.90), respectively. Among children with sepsis at admission (n=166), mortality was 16/46 (34.8%) in undernourished children and 19/120 (15.8%) in well-nourished children, corresponding to a crude RR of 2.20 (95% CI, 1.24–3.89) and an adjusted RR of 1.81 (95% CI, 1.03–3.18). The undernutrition × sepsis interaction was not statistically significant (P for interaction = .472), providing no evidence that the association between undernutrition and PICU mortality differed according to sepsis status ( **Supplementary Table S8).**

***Excluding Trauma/Toxicology Admissions***

After excluding children admitted with trauma or toxicology diagnoses, the analytical sample included 272 children. PICU mortality occurred in 22/215 (10.2%) well-nourished and 16/57 (28.1%) undernourished children (crude RR, 2.74; 95% CI, 1.55–4.87) (Supplementary Table 9). After adjusting for age, PIM-3 predicted mortality, chronic/comorbid conditions, and sepsis at admission, the association was attenuated and less precise (adjusted RR, 1.66; 95% CI, 0.94–2.93) (**Supplementary Table** **S9)**.

***PICU mortality by undernutrition status stratified by age***

Because of the marked age difference between the nutritional groups, we examined whether age modified the association between undernutrition and mortality. In age-stratified analyses, the adjusted risk ratio for undernutrition was 1.61 (95% CI 0.45–5.72) among children <1 year, 3.34 (95% CI 1.22–9.20) among children 1–5 years, and 1.23 (95% CI 0.45–3.35) among children >5 years (**Supplementary Table S11**). However, the formal joint test for heterogeneity across these age categories was not statistically significant (χ²=3.18, df=2, P=.203), and the continuous linear undernutrition × age interaction was likewise not significant (ratio of risk ratios per year 0.961, 95% CI 0.863–1.070, P=.464), indicating that the apparent elevation in the 1–5 year stratum should not be interpreted as statistical evidence of effect modification. A more flexible model using restricted cubic splines for age suggested a statistically significant nonlinear interaction (χ²=10.87, df=3, P=.013); however, this model included substantially more parameters relative to the 46 available deaths than the primary model (which itself had an events-per-variable ratio of only 9.2), and several age-nutrition strata contained few events. Therefore, we interpret this spline finding as an exploratory, hypothesis-generating signal rather than as confirmatory evidence of nonlinear effect modification.

Repeating these analyses within the propensity score-weighted (IPTW) framework yielded materially consistent results: stratum-specific adjusted risk ratios of 1.97 (95% CI 0.60–6.46), 3.10 (95% CI 1.08–8.89), and 1.22 (95% CI 0.39–3.87) for the <1 year, 1–5 year, and >5 year strata, respectively, with no significant linear (P=.662) or categorical (P=.313) interaction, and a similarly fragile spline interaction (P=.018) (**Supplementary Table S10**).

***Net Reclassification Improvement of undernutrition beyond PIM-3, sepsis, age, and chronic comorbidity***

Adding undernutrition to the base model (PIM-3 predicted mortality, sepsis, age, and chronic comorbid conditions) did not significantly improve reclassification. The event NRI was −0.109 (95% CI −0.375 to 0.354), and the non-event NRI was 0.481 (95% CI −0.193 to 0.670); the overall continuous NRI was 0.372 (95% CI −0.095 to 0.856) (**Supplementary Table S11a**). All three confidence intervals included zero, and the point estimate for events was directionally negative, indicating no statistically significant evidence that undernutrition improved individual-level risk classification in this cohort. Discrimination was essentially unchanged, with AUROC of 0.951 and 0.952 in the base and extended models, respectively (bootstrap 95% CI for the difference, −0.008 to 0.009), and the Brier score changed minimally (0.056 to 0.053; 95% CI for change, −0.010 to 0.000) ((**Supplementary Table S11b)**.

These findings indicate that, despite the robust independent association between undernutrition and mortality observed in our primary analyses (adjusted RR 2.78), adding undernutrition did not improve individual-level risk classification beyond PIM-3, sepsis, age, and chronic comorbidity in this cohort. This is consistent with the near-ceiling apparent discrimination of the base model (AUROC 0.951), which leaves limited room for any single additional covariate to improve classification and with the known instability of continuous NRI in small samples with a modest number of events. We interpret this null result as distinct from, rather than contradictory to, our primary finding: undernutrition appears to be an independently associated population-level risk factor for mortality, but this analysis does not provide evidence that it should be incorporated into individual-level risk-prediction tools without further validation in larger cohorts.

**E-value Analysis results**

The E-value for the primary adjusted association (aRR 2.78) was 5.00, and the lower 95% confidence limit (1.58) was 2.54. For the propensity score-weighted doubly robust estimate (weighted RR 2.59), the corresponding E-values were 4.62 and 2.01 (**Supplementary Table S12**), respectively. These values indicate that an unmeasured confounder would need to be associated with both undernutrition and mortality by risk ratios of approximately 4.6- to 5.0-fold each, above and beyond measured covariates, to fully explain the point estimate, and by 2.0- to 2.5-fold each to shift the lower confidence bound to the null.

**Ventilator-Free Days to Day 28: Distribution and Quantile Regression Sensitivity Analysis**

Because VFD-28 showed a marked ceiling effect (193/331 children [58.3%] had VFD-28=28), we performed median regression as a sensitivity analysis. This confirmed a statistically significant, directionally consistent association (adjusted median difference, −1.8 days; 95% CI, −6.9 to −0.2), although it was smaller in magnitude than the mean-based estimate, consistent with the mean being more heavily influenced by the cluster of zero values contributed by deaths, which were more frequent among undernourished children **(Supplementary Table S13**).

**Model Calibration and Predicted Risk Distribution (Figures 2, main manuscript and Supplementary Figure 1 and 2)** In the full cohort the mortality prediction demonstrated high apparent discrimination (AUC 0.952) and an optimism-corrected AUC of 0.946 after bootstrap internal validation; however, both values should be interpreted cautiously given the limited event count (n = 46, EPV 9.2), which creates conditions favorable for optimism, even with bootstrap correction. The optimism-corrected calibration slope was 0.855, indicating modest overfitting, which is typical of models developed with limited events. Decile-based calibration plots (**Figures 2, main manuscript, and Supplementary Figure 1 and 2**) showed good overall agreement between predicted and observed mortality across the risk range, with points generally tracking the 45-degree line of perfect calibration. Some departures were observed in the highest predicted-risk deciles, where the observed mortality (approximately 80%) was lower than the mean predicted mortality (approximately 85%), which was consistent with a calibration slope of <1. The ridge-penalized model showed essentially unchanged calibration on visual inspection, confirming that penalization primarily improved numerical stability rather than materially changing performance.

**The distribution of the predicted mortality probabilities (Supplementary Figure 1**) showed that the extreme predicted values were rare. Under the unpenalized model, the predicted risks ranged from 0.001 to 1.000, with the 99th percentile at 0.984, and only two observations exceeded 0.999. Under the ridge model, the predicted risks ranged from 0.009 to 1.000, with one observation exceeding 0.999 and a slightly less-extreme upper tail. Together, the calibration plots and risk distributions support that the model's discrimination and calibration were not driven by a small number of near-deterministic predictions and that the validation results were robust to the ridge penalization.

**Calibration of the prespecified PICU mortality prediction model (ridge-penalized logistic regression; Supplementary Figure 2)**. The model predicted mortality reasonably well across most of the risk spectrum, with the greatest deviation at higher predicted risk levels, where the model tended to underestimate mortality, consistent with the known miscalibration of PIM-3 in this high-burden setting. Ridge penalization was used during bootstrap internal validation to improve numerical stability in sparse bootstrap samples; its calibration pattern was similar to the unpenalized model (Figure 2), supporting robustness of model performance estimates.

**Supplementary Tables**

**Supplementary Table S1a**. **Chronic comorbidity categories by organ system according to nutritional status**

| **Comorbidity category**  **by organ system** | **Overall chronic comorbidity**  **subgroup (n = 90)** | **Undernourished**  **(n = 60)** | **Well nourished**  **(n = 271)** |
| --- | --- | --- | --- |
| Cardiac | 5 (5.6) | 1 (1.7) | 4 (1.5) |
| Neurologic | 15 (16.7) | 1 (1.7) | 14 (5.2) |
| Respiratory | 11 (12.2) | 2 (3.3) | 9 (3.3) |
| Hematology-oncology | 28 (31.1) | 10 (16.7) | 18 (6.6) |
| Renal | 4 (4.4) | 0 (0.0) | 4 (1.5) |
| Other | 27 (30.0) | 6 (10.0) | 21 (7.7) |

Data are presented as numbers (percentages). The percentages of undernourished and well-nourished children were calculated using all children in each nutritional group as denominators. The percentages for the overall chronic comorbidity subgroup were calculated using children with chronic comorbidities as denominators. The other category included trauma, toxicologic conditions, diabetic ketoacidosis, rheumatologic disorders, gastrointestinal conditions, and other miscellaneous diagnoses according to the investigator-defined classification. Four cases of renal comorbidity were classified separately, all in well-nourished children. The overall distribution of comorbidity categories did not differ according to nutritional status (Fisher’s exact test, P = .32).

**Supplementary Table S1b. Anthropometric deficits at PICU admission by age group**

| **Measure / metric** | **Prevalence of anthropometric deficits, n/N (%)** | | | |
| --- | --- | --- | --- | --- |
|  | **<5 years** | **5-18 years** | | **Overall** |
| Underweight  (Weight for age < − 2SD) | 58/181 (32.0%) | 2/150 (1.3%) | | 60/331 (18.1%) |
| Severe Underweight  (Weight for age < − 3SD) | 28/181 (15.5%) | 0/150 (0.0%) | | 28/331 (8.5%) |
| Stunting  (Height for age < − 2SD) | 53/181 (29.3%) | 23/150 (15.3%) | | 76/331 (23.0%) |
| Severe stunting  (Height for age < − 3SD | 16/181 (8.8%) | 9/150 (6.0%) | | 25/331 (7.6%) |
| Undernutrition  (Weight for Height/length for age <− 2SD) | 49/181 (27.1%) | 11/150 (7.3%) | | 60/331 (18.1%) |
| Severe undernutrition  (Weight for Height/length for age <− 3SD) | 24/181 (13.3%) | | 5/150 (3.3%) | 29/331 (8.8%) |

Data are presented as numbers/total number. (%). Underweight was defined as a weight-for-age z score < −2SD, and severe underweight was defined as < −3SD. Stunting was defined as a length/height-for-age z-score < −2SD and severe stunting as < −3SD. Undernutrition was defined using an age-appropriate composite anthropometric indicator: weight-for-length/height z-score for children younger than 5 years and body mass index (BMI)-for-age z-score for children aged 5–18 years. Severe undernutrition was defined as a z-score of < −3SD.

**Supplementary Table S1c. Anthropometric summary of the full cohort according to undernutrition status, median (IQR)**

| **Measure / metric** | **Overall** | **Undernourished** | **Well-nourished** |
| --- | --- | --- | --- |
| Weight at admission, kg | 13.20 (8.50 to 25.00) | 8.00 (6.80 to 11.05) | 16.00 (10.00 to 28.20) |
| Length/height, cm | 96.50 (75.00 to 128.00) | 78.50 (73.50 to 96.25) | 105.00 (77.50 to 132.00) |
| Composite BMI-for-age or Weight-for-Height z score | −0.52 (-1.63 to 0.50) | −2.89 (−3.80 to −2.29) | −0.14 (−0.81 to 0.66) |
| Weight-for-age z score | −0.83 (−1.62 to −0.26) | −2.60 (−3.11 to −1.67) | −0.64 (−1.21 to −0.11) |
| Height/length-for-age z score | −1.02 (−1.89 to −0.19) | −0.88 (−2.11 to −0.03) | −1.04 (−1.87 to −0.20) |

Data are presented as median (IQR). The columns show the overall cohort, undernourished children, and well-nourished children. Weight-for-age and length/height-for-age z-scores for the full cohort were calculated using the World Health Organization standards for children younger than 5 years and Indian Academy of Pediatrics references for children aged 5–18 years. The composite anthropometric z-score was the weight-for-length/height z-score for children younger than 5 years and the body mass index-for-age z-score for children aged 5–18 years.

Categorical anthropometric deficits are presented as No./total No. (%), and continuous anthropometric variables are presented as median (IQR)

**Supplementary Table S1d. Bootstrap Validation for the Adjusted Relative Risk of PICU Mortality Associated with Undernutrition**

| **Section** | **Parameter** | **Value** |
| --- | --- | --- |
| Bootstrap settings | Bootstrap replicates | 1000 |
| Bootstrap settings | Random seed | 20251221 |
| Effect estimate (Adjusted RR***^a^*** for undernutrition) | Original point estimate (adjusted RR) | 2.78 |
|  | Bootstrap median | 2.51 |
|  | Bootstrap mean | 2.64 |
| 95% confidence intervals | Standard 95% CI (original adjusted model)† | 1.58–4.91 |
|  | Bootstrap percentile 95% CI | 1.20–4.83 |
|  | Bootstrap BCa 95% CI | 1.48–6.10 |
| Robustness across resamples | Bootstrap samples with Wald 95% CI excluding 1.0, No. (%) | 837/1000 (83.7%) |

Abbreviations: BCa, bias-corrected and accelerated.

***^a^***The undernutrition effect is the adjusted relative risk (RR) of PICU mortality comparing undernourished vs. well-nourished children from the prespecified modified Poisson model with robust standard errors (adjusted for age, PIM-3 score, baseline sepsis, and chronic comorbidities). †The “standard CI” is the model-based Wald 95% CI from the original cohort. Bootstrap summaries and CIs were based on 1,000 nonparametric resamples with replacement. “Bootstrap samples with Wald 95% CI excluding 1.0” is the proportion of bootstrap refits in which the resample-specific Wald 95% CI for the RR did not include 1.0.

**Supplementary Table S1e. Cause-Specific Cox Sensitivity Analysis for Time to Alive PICU Discharge**

| **Outcome** | **Model** | **Effect estimate**  **(95% CI)** | **P value** | **Interpretation** |
| --- | --- | --- | --- | --- |
| Alive PICU discharge | Cause-specific Cox proportional hazards model (death treated as competing event) | HR 0.58 (0.43 to 0.78) | <.001 | Lower hazard of alive discharge, consistent with longer PICU stay/slower recovery among undernourished children |

**Abbreviations:** CI, confidence interval; HR, hazard ratio; PICU, pediatric intensive care unit.

The model was adjusted for age, PIM-3 predicted mortality at admission, chronic/comorbid conditions, and sepsis at admission.

The cause-specific Cox model estimated the instantaneous hazard of alive PICU discharge among children who were still alive and not yet discharged at each time point; a lower hazard ratio indicated slower recovery and longer PICU stay.

**Supplementary Table S2a. Absolute Standardized Mean Differences (|SMD|) for propensity score covariates before and after trimming, stabilized inverse probability of treatment weighting (IPTW).**

| **Covariate** | **Absolute SMD, unweighted** | **Absolute SMD, IPTW (trimmed)** |
| --- | --- | --- |
| Age, years | 0.821 | 0.397 |
| PIM-3 predicted probability of death | 0.191 | 0.143 |
| Chronic or comorbid condition | 0.164 | 0.054 |
| Sepsis at admission | 0.699 | 0.188 |
| Female sex | 0.167 | 0.012 |
| Maternal education (graduate or postgraduate) | 0.235 | 0.007 |

**Abbreviations**: IPTW, inverse probability of treatment weighting; PIM-3, Pediatric Index of Mortality 3; PS, propensity score; SMD, standardized mean difference. The absolute SMDs compared the covariate balance between undernourished and well-nourished children before and after weighting; values closer to 0 indicate better balance. Stabilized IPTW were based on the marginal exposure prevalence divided by each individual PS and were trimmed at the 1st and 99th percentiles, then rescaled to have a mean of 1; a prespecified cap of 20 was applied if needed.

| **Supplementary Table S2b. Propensity Score Model Diagnostics and Inverse Probability of Treatment Weighting (IPTW) Diagnostics for Undernutrition Exposure.** | | |
| --- | --- | --- |
| **Metric** | **Parameter** | **Value** |
| Propensity score model AUROC (c-statistic) |  | 0.803 |
| AUROC 95% CI |  | 0.746 to 0.860 |
| Exposure prevalence (undernourished) |  | 18.1% |
| Weighting approach |  | Stabilized IPTW (ATE) |
| Trimming rule |  | 1st and 99th percentiles of stabilized weights (0.290 to 2.032) |
| Trimmed observations (n) |  | 8 |
| Trimmed observations (%) |  | 2.42% |
| Weight cap |  | Rescaled to mean 1 then capped at 20 |
| Weights capped at 20 (n) |  | 0 |
| Weights capped at 20 (%) |  | 0.00% |
| Raw stabilized weight percentiles | minimum | 0.253 |
|  | P1 | 0.290 |
|  | P5 | 0.404 |
|  | P50 | 0.895 |
|  | P95 | 1.640 |
|  | P99 | 2.032 |
|  | maximum | 11.102 |
| Effective sample size (ESS) |  | 298.7 |
| ESS as % of actual n |  | 90.2% |
| Covariate balance summary (absolute SMD) |  |  |
| Max \|SMD\| | Pre-IPTW weighting | 0.821 |
|  | Post-IPTW weighting | 0.397 |
| Mean \|SMD\| | Pre-IPTW weighting | 0.373 |
|  | Post-IPTW weighting | 0.133 |

Abbreviations: ATE, average treatment effect; AUROC, area under the receiver operating characteristic curve; IPTW, inverse probability of treatment weighting; PS, propensity score; SMD, standardized mean difference; P, percentile. The PS for undernutrition was estimated using multivariable logistic regression analysis. Stabilized IPTW (ATE) was trimmed at the 1st and 99th percentiles and rescaled to have a mean of 1; a prespecified cap of 20 was applied, if needed. “Observations affected by trimming” denotes the number of participants whose weights were in the trimmed tails of the weight distributions.

**Supplementary Table S3: Propensity Score–Weighted, Doubly Robust Sensitivity Analysis of the Association Between Undernutrition and PICU Mortality Using Trimmed, Stabilized Inverse Probability of Treatment Weighting (IPTW)**

| **PS Covariate Adjustment Set** | **n** | **Weighted RR (95% CI), Undernourished vs. Well-nourished** | **P value** |
| --- | --- | --- | --- |
| Age, PIM-3 (predicted probability of death), Chronic/comorbid condition, Sepsis at admission, Female sex, Maternal education( Graduate or postgraduate) | 331 | 2.59 (1.34–5.03) | .005 |

**Abbreviations**: CI, confidence interval; IPTW, inverse probability of treatment weighting; PICU, pediatric intensive care unit; PIM-3, Pediatric Index of Mortality 3; PS, propensity score; RR, relative risk. Estimates were obtained from a doubly robust model using trimmed, stabilized IPTW derived from the PS model, including age, PIM-3 predicted probability of death, chronic/comorbid condition, sepsis at admission, female sex, and maternal education (graduate/postgraduate). The weighted outcome model was additionally adjusted for age, PIM-3 predicted probability of death, chronic/comorbid conditions, and sepsis at admission.

**Supplementary Table S4. Sensitivity Analyses with Alternative Model Specifications for the Association Between Undernutrition and PICU Mortality**

| **Model** | **n** | **No. of Covariates** | **Description** | **Adjusted RR (95% CI)** |
| --- | --- | --- | --- | --- |
| Primary model (continuous PIM-3 ) | 331 | 5 | All children; PIM-3 per SD increase; adjusted for age, chronic/comorbid conditions, and sepsis at admission. | 2.78 (1.58–4.91) |
| PIM-3 tertiles (functional form; 5–variable)***^a^*** | 331 | 5 | The primary model was repeated with PIM-3 entered as tertiles (T1, T2, and T3) instead of continuous per SD. | 2.28 (1.36–3.84) |
| PIM-3 tertiles (3–variable model)***^b^*** | 331 | 3 | The model included nutritional status (undernourished vs. well-nourished), PIM-3 tertiles (T1, T2,and T3), and sepsis at admission. | 1.93 (1.23–3.03) |
| Exclude extreme PIM-3 (>=97.5th percentile) | 322 | 5 | The primary model was repeated after excluding children with extreme illness severity, defined a priori as PIM-3 >= 10.25 (97.5th percentile). | 1.89 (1.08–3.31) |
| Winsorize PIM-3 (cap at 95th percentile) | 331 | 5 | The primary model was repeated after winsorizing PIM-3 by capping values above 8.55 (95th percentile) while retaining all children. | 1.52 (1.02–2.34) |
| Excluding obese children | 324 | 5 | The primary model was repeated after excluding overweight or obese children, defined as BMI or weight-for-height z-score > +2. | 2.72 (1.54–4.78) |

Abbreviations: BMI, body mass index; CI, confidence interval; PICU, pediatric intensive care unit; PIM-3, Pediatric Index of Mortality 3; RR, relative risk; SD, standard deviation. All estimates were obtained from prespecified modified Poisson regression models with a log link and robust standard error.

***^a^***5-variable model included undernutrition (undernourished vs. well-nourished) and covariates age, PIM-3 predicted probability of death (per 1-SD increase, or winsorized as specified), chronic/comorbid condition, and sepsis at admission.

***^b^***3-variable model included undernutrition, PIM-3 predicted mortality tertiles (T1–T3), and sepsis at admission. PIM-3 tertiles (T1–T3) represent ordered categories of PIM-3 predicted mortality derived from the analytic cohort (T1 lowest, T3 highest). Extreme-severity sensitivity analyses used prespecified percentile-based rules computed within the analytic cohort: exclusion of PIM-3 ≥10.25 (97.5th percentile) and winsorization by capping PIM-3 at 8.55 (95th percentile).

**Supplementary Table S5.** **Mortality by Undernutrition Status Stratified by PIM-3 Tertile**

| **PIM-3 predicted mortality tertile** | **Undernourished, deaths/total (%)** | **Well-nourished, deaths/total (%)** | **RR*^a^* (95% CI)** | **P value** |
| --- | --- | --- | --- | --- |
| T1 (lowest PIM-3, predicted mortality - Low) | 0/19 (0.0) | 2/102 (2.0) | Not estimable | — |
| T2 (middle PIM-3, predicted mortality - moderate) | 0/15 (0.0) | 0/92 (0.0) | Not estimable | — |
| T3 (highest PIM-3, predicted mortality - high) | 18/26 (69.2) | 26/77 (33.8) | 2.05 (1.37–3.07) | < .001 |
| P for interaction (undernutrition × PIM-3 tertile) |  |  |  | .889 |

**Footnote:** PIM-3 predicted mortality tertiles were derived from the observed distribution within the analytic cohort (n = 331): tertile 1 (T1, ≤ 0.2%, n = 121), tertile 2 (T2, 0.3–2.9%, n = 107), and tertile 3 (T3, > 2.9%, n = 103). ***^a^***Risk ratios comparing undernourished versus well-nourished children were estimated using log-transformed Wald methods when estimable. RRs are reported as "Not estimable" when either exposure group had zero deaths within a tertile.

The P value for interaction was obtained from a likelihood ratio test using Firth penalized logistic regression comparing models with and without the undernutrition × tertile interaction term.(The Firth method was used due to sparse events and separation).

**Supplementary Table S6. Dose-Response Analysis Across Undernutrition Severity**

| **Nutritional Status*^a^*** | **Deaths/Total (%)** | **Crude RR*^b^***  **(95% CI)** | **Adjusted RR*^c^* (95% CI)** | **P value** |
| --- | --- | --- | --- | --- |
| Normal nutrition (z-score ≥ −2SD) | 28/271 (10.3) | 1 [Reference] | 1 [Reference] | — |
| Moderate undernutrition (z-score < −2SD to −3SD) | 10/31 (32.3) | 3.12 (1.68–5.80) | 1.75 (1.12–2.74) | .014 |
| Severe undernutrition (z-score < −3SD) | 8/29 (27.6) | 2.67 (1.34–5.30) | 1.51 (0.96–2.39) | .076 |
| P for linear trend | — | — | — | .023 |

**Abbreviations**: CI, confidence interval; RR, relative risk.

***^a^*** Nutritional status was categorized using BMI-for-age or weight-for-height z-scores according to the WHO/IAP standards.

*^b^*Relative risks and 95% CIs were estimated using Poisson regression models with a log link and robust standard errors. ***^c^***Adjusted for Pediatric Index of Mortality 3 (PIM-3) score in tertiles and presence of sepsis at admission.

**Supplementary Table S7. Standardized Mortality Ratios Overall and by Nutritional Status**

| **Category** | **Observed Deaths** | **Expected Deaths (PIM-3)** | **SMR (95% CI)** |
| --- | --- | --- | --- |
| Overall | 46 | 9.94 | 4.63 (3.39–6.17) |
| Undernourished | 18 | 2.28 | 7.90 (4.68–12.49) |
| Well–nourished | 28 | 7.66 | 3.65 (2.43–5.28) |

**Abbreviations**: CI, confidence interval; PIM-3, Pediatric Index of Mortality 3; SMR, standardized mortality ratio.

Expected deaths were computed as the sum of individual predicted probabilities from PIM-3 (PIM-3 predicted mortality was recorded as a percentage and converted to a probability by dividing by 100). SMR 95% confidence intervals (CIs) were calculated using the chi-square method for the Poisson count of observed deaths.

**Supplementary Table S8. PICU Mortality by Undernutrition Status Stratified by Sepsis at Admission**

| **Subgroup** | **n** | **Deaths in Undernourished n/N (%)** | **Deaths in Well-nourished n/N (%)** | **Crude RR**  **(95% CI)** | **Adjusted RR*^a^***  **(95% CI)** |
| --- | --- | --- | --- | --- | --- |
| No sepsis | 165 | 2 / 14  (14.3%) | 9 / 151 (6.0%) | 2.40 (0.57–10.03) | 4.51 (1.37–14.90) |
| Sepsis present | 166 | 16 / 46  (34.8%) | 19 / 120 (15.8%) | 2.20 (1.24–3.89) | 1.81 (1.03–3.18) |
| P for interaction (undernutrition x sepsis) | — | — | — | — | .472 |

**Abbreviations:** CI, confidence interval; PICU, pediatric intensive care unit; RR, risk ratio. Data are presented as deaths/total (percentage). Crude RRs compare the risk of PICU mortality in undernourished versus well-nourished children within the sepsis strata at admission.

***^a^***Adjusted RRs were obtained from modified Poisson regression with robust standard errors, fitted separately within each sepsis stratum, and adjusted for age (years), PIM-3 predicted mortality, and chronic/comorbid conditions. P for interaction was obtained from the undernutrition × sepsis term in the adjusted modified Poisson model fitted to the full cohort.

**Supplementary Table S9. Association Between Undernutrition and PICU Mortality After Excluding Trauma/Toxicology Admissions**

| **Nutritional status** | **No. of patients** | **No. of deaths** | **Crude RR**  **(95% CI)** | **Adjusted RR*^a^***  **(95% CI)** |
| --- | --- | --- | --- | --- |
| Well nourished | 215 | 22 | 1.00 (reference) | 1.00 (reference) |
| Undernourished | 57 | 16 | 2.74 (1.55–4.87) | 1.66 (0.94–2.93) |

Abbreviations: CI, confidence interval; PICU, pediatric intensive care unit; RR, risk ratio. Children with trauma or toxicology diagnoses were excluded. Crude RRs were calculated using 2×2 tables. ***^a^***Adjusted RRs were estimated using modified Poisson regression model with a log link and a robust standard error, adjusting for age (years), PIM-3 predicted mortality at admission, chronic/comorbid conditions, sepsis at admission.

**Supplementary Table S10. Age Distribution, Stratified Mortality, and Age-Interaction Analyses for the Undernutrition-Mortality Association**

| **Panel A: Age and crude mortality by nutritional status and age category** | | | | |
| --- | --- | --- | --- | --- |
| **Age category** | **Nutritional status** | **N** | **% within category** | **Deaths, n/N (%)** |
| <1 year | Well-nourished | 47 | 71.2 | 3/47(6.4) |
|  | Undernourished | 19 | 28.8 | 6/19 (31.6) |
| 1–5 years | Well-nourished | 88 | 73.9 | 7/88 (8) |
|  | Undernourished | 31 | 26.1 | 9/31(29.0) |
| >5 years | Well-nourished | 136 | 93.2 | 18/136 (13.2) |
|  | Undernourished | 10 | 6.8 | 3/10 (30) |
| **Panel B: Age-stratified and interaction analyses (unweighted and IPTW-weighted)^a^** | | | | |
| **Analysis** | **Unweighted aRR (95% CI)** | **P value** | **IPTW aRR (95% CI)** | **P value** |
| Age <1 year | 1.61 (0.45–5.72) | .460 | 1.97 (0.60–6.46) | .264 |
| Age 1–5 years | 3.34 (1.22–9.20) | .019 | 3.10 (1.08–8.89) | .035 |
| Age >5 years | 1.23 (0.45–3.35) | .684 | 1.22 (0.39–3.87) | .733 |
| Overall model (age as covariate)**^b^** | 2.78 (1.58–4.91) | <.001 | 2.59 (1.34–5.03) | .005 |
| Linear age interaction (RR per year)**^c^** | 0.961 (0.863–1.070) | .464 | 0.969 (0.841–1.116) | .662 |
| Categorical age interaction**^d^** | χ²=3.18, df=2 | .203 | χ²=2.32, df=2 | .313 |
| Spline age interaction**^s^** | χ²=10.87, df=3 | .013 | χ²=10.02, df=3 | .018 |

Abbreviations: aRR, adjusted risk ratio; CI, confidence interval; IPTW, inverse probability of treatment weighting; RR, risk ratio.

All models were adjusted for PIM-3 predicted mortality, sepsis at admission, and chronic comorbid conditions. IPTW models additionally used stabilized trimmed weights, as described in **Supplementary Tables S2a and S2b**.

**^a^**Stratified analyses estimated the undernutrition-mortality association separately within each age stratum; P values reflect the test of each stratum-specific risk ratio against the null (RR=1), not a test of heterogeneity between strata.

**^b^**Reproduces the primary adjusted model (Table 2, main manuscript; Supplementary Table S3) for reference.

**^c^**The ratio of risk ratios reflects the multiplicative change in the undernutrition-associated risk ratio per year of age.

**^d^**Joint Wald test for the undernutrition × age-category interaction term; this, not the individual stratum-specific P values in the rows above, is the appropriate test for statistical evidence of effect modification by age category.

**^e^**Joint Wald test for the undernutrition × restricted cubic spline age interaction.

Given the limited number of deaths (n=46) relative to the additional parameters required for spline modeling and the lack of significance of the linear and categorical interaction tests, this result should be interpreted as exploratory and hypothesis-generating rather than confirmatory.

**Supplementary Table S11a. Net Reclassification Improvement of Undernutrition Beyond PIM-3, Sepsis, Age, and Chronic Comorbidity**

**[***Models compared (n = 331; 46 deaths in both models****^a^****): Base model: PIM-3 predicted mortality + sepsis + age + chronic comorbidity. Extended model: Base model + undernutrition status]*

| **Reclassification component** | **Estimate (95% CI)^b^** | **Among deaths (n = 46)** | **Among survivors (n = 285)** |
| --- | --- | --- | --- |
| Event NRI | -0.109 (-0.375 to 0.354) | Risk increased: 43.5%  Risk decreased: 54.3% | — |
| Non-event NRI | 0.481 (-0.193 to 0.670) | — | Risk decreased: 74.0%  Risk increased: 26.0% |
| Overall continuous NRI | 0.372 (-0.095 to 0.856) | — | — |

Abbreviations: CI, confidence interval; NRI, net reclassification improvement; PIM-3, Pediatric Index of Mortality 3.

**^a^**Predictions for both models were generated from logistic regression using apparent (within-sample, non-optimism-corrected) predicted probabilities.

**^b^**Event NRI reflects the net proportion of children who died and were assigned a higher predicted risk by the extended versus base model; non-event NRI reflects the net proportion of survivors assigned a lower predicted risk. Overall, the continuous NRI is the sum of the event and non-event NRI. Ties were classified as neither improved nor worsened. Ninety-five percent CIs were obtained from 1,000 bootstrap resamples. All three intervals included zero, indicating no statistically significant evidence of improved reclassification; the event NRI point estimate was directionally unfavorable (more deaths reclassified to lower rather than higher risk).

**Supplementary Table S11b. Discrimination Comparison Between Base and Extended Models**

| **Metric** | **Base model** | **Extended model** | **Difference** | **Bootstrap 95% CI for difference** |
| --- | --- | --- | --- | --- |
| AUROC (c-statistic) | 0.951 | 0.952 | 0.001 | -0.008 to 0.009 |
| Brier score | 0.056 | 0.053 | -0.003 | -0.010 to 0.000 |

Abbreviations: AUROC, area under the receiver operating characteristic curve; CI, confidence interval.

Both models showed an apparent calibration intercept of 0 and a calibration slope of 1 by construction, as both were fit by maximum likelihood and evaluated on the same data used for fitting; this is mathematically guaranteed for in-sample logistic regression and is not reported as it provides no information about relative model performance. Discrimination was materially unchanged with the addition of undernutrition, consistent with the near-ceiling apparent AUROC of the base model.

**Supplementary Table S12. E-Value Analysis for Unmeasured Confounding**

| **Analysis** | **RR (95% CI)** | **E-value, point estimate** | **E-value, lower CI** |
| --- | --- | --- | --- |
| Primary adjusted model | 2.78 (1.58-4.91) | 5.00 | 2.54 |
| IPTW doubly robust model | 2.59 (1.34-5.03) | 4.62 | 2.01 |

Abbreviations: CI, confidence interval; IPTW, inverse probability of treatment weighting; RR, risk ratio.

E-values represent the minimum risk ratio that an unmeasured confounder would need to have with both undernutrition and PICU mortality, conditional on measured covariates, to fully explain the observed association (point estimate) or shift the confidence interval to include the null (lower CI bound). E-values quantify sensitivity to unmeasured confounding only; they do not address potential exposure misclassification or reverse causation, which are considered separately in the limitations.

**Supplementary Table S13. Ventilator-Free Days to Day 28: Distribution and Quantile Regression Sensitivity Analysis**

| **Panel A. Distribution by nutritional status** | | | | | |
| --- | --- | --- | --- | --- | --- |
| **Nutritional status** | **n** | **Mean (SD)** | **Median (IQR)** | **VFD-28 = 28, n (%)** | **VFD-28 = 0, n (%)** |
| Well-nourished | 271 | 23.9 (8.7) | 28.0 (26.0-28.0) | 173 (63.8) | 29 (10.7) |
| Undernourished | 60 | 17.5 (12.0) | 23.5 (0.0-28.0) | 20 (33.3) | 18 (30.0) |
| **Panel B. Adjusted association between undernutrition and VFD-28^a^** | | | | | |
| **Method** | | | **Estimate (95% CI)** | | **P** |
| Adjusted median regression (tau = 0.50)^b^ | | | -1.8 days (-6.9 to -0.2) | | .014 |
| Adjusted linear regression (mean)**^c^** | | | -3.8 days (-6.1 to -1.4) | | .002 |

Abbreviations: CI, confidence interval; VFD-28, ventilator-free days to day 28.

**^a^**Both models were adjusted for age, PIM-3 predicted mortality, chronic/comorbid conditions, and sepsis at admission; VFD-28 was calculated for all 331 children, constrained to 0-28 days.

**^b^**Bootstrap 95% CI from 1,000 resample. Median regression is less sensitive than the mean-based model to the cluster of zero values contributed by the deaths.

**^c^**Reproduces the primary linear regression estimate (Table 4) for comparison.

Both methods indicated fewer ventilator-free days among undernourished children, with the smaller median-based estimate reflecting reduced sensitivity to the higher proportion of deaths (zero values) in this group rather than a contradictory finding.

**Supplementary Figure 1.** Distribution of Predicted PICU Mortality Risk

**Abbreviations**: CV, cross-validation.

**Caption**: Histograms of individual predicted probabilities of PICU mortality from the prespecified logistic regression model, shown separately for the standard unpenalized model (pink) and ridge-penalized model with cross-validation-selected penalty (teal). The figure shows the spread of predicted risk across the cohort and evaluates whether apparent discrimination and calibration might be disproportionately influenced by a small number of extreme predicted probabilities (near 0 or 1), which can occur with quasi-complete separation when sparse covariate patterns yield near-deterministic predictions.

The main message of this figure is that extreme predicted values were rare: under the unpenalized model, only two observations exceeded a predicted probability of 0.999, and the ridge model produced a slightly less extreme upper tail. The close agreement between the two distributions confirms that ridge penalization improved numerical stability without materially altering the predicted risk distribution and that model discrimination and calibration were not driven by a small number of near-deterministic predictions.

**Footnote**: Ridge logistic regression was performed with the penalty parameter (λ) selected via 10-fold cross-validation to optimize the predictive performance while reducing overfitting. This model was used during bootstrap internal validation only; primary inference was based on modified Poisson regression, as described in the Methods section.

**Supplementary Figure 2.** Calibration of the prespecified PICU mortality prediction model (ridge-penalized logistic regression). **Abbreviations**: CV, cross-validation.

Patients were grouped into deciles according to their predicted mortality risks. Within each decile, the observed proportion of deaths (y-axis) was plotted against the mean predicted probability of death (x-axis). The dashed diagonal line represents perfect calibration, where the predicted and observed mortality rates are equal. Points above the line indicate that the model underestimated the mortality risk (more deaths observed than predicted), and points below indicate overestimation. The main message of this figure is that the model predicted mortality reasonably well across most of the risk spectrum, with the greatest deviation at higher predicted risk levels, where the model tended to underestimate mortality, consistent with the known miscalibration of PIM-3 in this high-burden setting. Ridge penalization was used during bootstrap internal validation to improve numerical stability in sparse bootstrap samples; its calibration pattern was similar to the unpenalized model (Figure 2), supporting robustness of model performance estimates.

**Supplementary Figure 3. Adjusted mortality risk according to age and nutritional status.**

**Caption:** Predicted PICU mortality risk across age, shown separately for well-nourished and undernourished children using a modified Poisson regression model with restricted cubic spline modeling of age, adjusted for PIM-3 predicted mortality, sepsis at admission and chronic comorbid conditions. Shaded bands represent 95% confidence intervals. Points show observed crude mortality within age categories by nutritional status, with point size proportional to the number of children in each stratum.

This figure corresponds to the exploratory spline-based age-by-nutrition interaction analysis presented in Supplementary Table S10. This was provided for visual transparency regarding the shape of the age-mortality relationship within each nutritional group. The spline interaction test reached nominal statistical significance (P=.013); however, the confidence intervals were wide across the full age range, particularly at the extremes of age, where undernourished children were few. Accordingly, this figure and the corresponding interaction analysis should be interpreted as hypothesis-generating rather than as confirmatory evidence of nonlinear effect modification by age, and the primary conclusion that the undernutrition-mortality association was consistent across age strata based on linear and categorical interaction tests (both P>.20) is unchanged.

**Footnote**: Restricted cubic splines were fitted with knots placed at the 5th, 35th, 65th, and 95th percentiles of the age distribution. The spline interaction model includes substantially more parameters than the primary model relative to the number of deaths (n=46) and should be interpreted accordingly.

**STROBE Checklist — Observational Study (9)**

**Study Title:** Association Between Admission Undernutrition and Mortality Rates and Adverse Outcomes in a Pediatric Intensive Care Unit in India: A Prospective Cohort Study

**Study Design:** Prospective Cohort Study

| **Item No.** | **STROBE Recommendation** | **Location in Manuscript** | **Page/Section** |
| --- | --- | --- | --- |
| 1 | Title and Abstract | (a) Indicate the study's design with a commonly used term in the title or the abstract  (b) Provide in the abstract an informative and balanced summary of what was done and what was found | Title, Page 1  Abstract, Page 4 |
| 2 | Background/rationale | Explain the scientific background and rationale for the investigation being reported | Introduction  Page 2 |
| 3 | Objectives | State specific objectives, including any prespecified hypotheses | Introduction  Page 2 |
| 4 | Study design | Present key elements of study design early in the paper | Methods;  Page 2 |
| 5 | Setting | Describe the setting, locations, and relevant dates, including periods of recruitment, exposure, follow-up, and data collection | Methods;  Page 2 |
| 6 | Participants | (a) Cohort study—Give the eligibility criteria, and the sources and methods of selection of participants. Describe methods of follow-up  (b) Cohort study—For matched studies, give matching criteria and number of exposed and unexposed | Methods;  Page 3 |
| 7 | Variables | Clearly define all outcomes, exposures, predictors, potential confounders, and effect modifiers. Give diagnostic criteria, if applicable | Methods; Page 3 |
| 8 | Data sources/measurement | For each variable of interest, give sources of data and details of methods of assessment (measurement). Describe comparability of assessment methods if there is more than one group | Methods;  Page 3-4  Supplementary Methods |
| 9 | Bias | Describe any efforts to address potential sources of bias | Sensitivity and exploratory analysis in Methods section, Statistical Analysis; Page 4 -5 ,and Supplementary Methods |
| 10 | Study size | Explain how the study size was arrived at | Methods, page 4 and  Supplementary methods |
| 11 | Quantitative variables | Explain how quantitative variables were handled in the analyses. If applicable, describe which groupings were chosen and why | Methods - Definitions: z-score categorization; page 3 |
| 12 | Statistical methods | (a) Describe all statistical methods, including those used to control for confounding  (b) Describe any methods used to examine subgroups and interactions  (c) Explain how missing data were addressed  (d) Cohort study—If applicable, explain how loss to follow-up was addressed  (e) Describe any sensitivity analyses | Methods - Statistical Analysis; Page 4-5 and Supplementary methods: all methods |
| 13 | Participants | (a) Report numbers of individuals at each stage of study—eg numbers potentially eligible, examined for eligibility, confirmed eligible, included in the study, completing follow-up, and analysed  (b) Give reasons for non-participation at each stage  (c) Consider use of a flow diagram | Results paragraph 1, page 5.  Figure 1: Participant flow with reasons. |
| 14 | Descriptive data | (a) Give characteristics of study participants (eg demographic, clinical, social) and information on exposures and potential confounders  (b) Indicate number of participants with missing data for each variable of interest  (c) Cohort study—Summarise follow-up time (eg, average and total amount) | a) Results paragraph 1, page 5 , and  Table 1  b) Methods; page 4 |
| 15 | Outcome data | Cohort study—Report numbers of outcome events or summary measures over time | Results paragraphs 1 : page 5  outcome data by exposure; Tables 1, |
| 16 | Main results | (a) Give unadjusted estimates and, if applicable, confounder-adjusted estimates and their precision (eg, 95% confidence interval). Make clear which confounders were adjusted for and why they were included  (b) Report category boundaries when continuous variables were categorized  (c) If relevant, consider translating estimates of relative risk into absolute risk for a meaningful time period | Results paragraphs : page 6-7  outcome data by exposure; Tables 2, and 4 |
| 17 | Other analyses | Report other analyses done—eg analyses of subgroups and interactions, and sensitivity analyses | Supplemental Methods and Results  Supplementary Tables **S**1-**S**13: sensitivity and exploratory analyses |
| 18 | Key results | Summarise key results with reference to study objectives | Discussion paragraph 1: summary of key findings; page 8 |
| 19 | Limitations | Discuss limitations of the study, taking into account sources of potential bias or imprecision. Discuss both direction and magnitude of any potential bias | Discussion: Limitations section; page 11-12. |
| 20 | Interpretation | Give a cautious overall interpretation of results considering objectives, limitations, multiplicity of analyses, results from similar studies, and other relevant evidence. | Discussion: interpretation of biological mechanisms; Pages 8-12. |
| 21 | Generalisability | Discuss the generalisability (external validity) of the study results | Discussion: implications for practice and policy and Limitations section; page 11-12 |
| 22 | Funding | Give the source of funding and the role of the funders for the present study and, if applicable, for the original study on which the present article is based | Funding and Acknowledgments section; page 13-14 |

Notes**:** This checklist confirms that the manuscript addresses all STROBE requirements for reporting prospective cohort study. The page numbers refer to the submitted manuscript. All 22 STROBE items were adequately addressed in this manuscript.

**References**

1 WHO Multicentre Growth Reference Study Group. WHO Child Growth Standards: Methods and Development. Geneva: World Health Organization (2006)

2 Indian Academy of Pediatrics Growth Charts Committee, Khadilkar V, Yadav S, Agrawal KK, Tamboli S, Banerjee M et al. Revised IAP growth charts for height, weight and body mass index for 5- to 18-year-old Indian children. Indian Pediatr (2015) 52:47-55. doi: [10.1007/s13312-015-0566-5](https://doi.org/10.1007/s13312-015-0566-5)

3 Ministry of Health and Family Welfare, Government of India (2019-21) India Report. National Family Health survey (NFHS-5). DHS Program. <https://dhsprogram.com/pubs/pdf/FR375/FR375.pdf> [Accessed January 2, 2026]

4 Amirjani S, Ahmadizadeh N, Behzad A, Dadashi-Noshahr Y, Vahdat Shariatpanahi Z. Undernutrition and 60-day mortality in critically ill children with respiratory failure: A prospective cohort study. BMC Pediatr (2023) 23:271. doi: [10.1186/s12887-023-04087-x](https://doi.org/10.1186/s12887-023-04087-x)

5 Agrawal A, Sharma S, Janjua D, Jadon G, Chanchlani R, Dsouza V. Impact of nutritional status on the mortality and clinical outcomes of children admitted to the pediatric intensive care unit: A systematic review and meta-analysis. Clin Nutr (2025) 51:28-39. doi: [10.1016/j.clnu.2025.06.002](https://doi.org/10.1016/j.clnu.2025.06.002)

6 Toh SC, Ong C, Sultana R, Kirk AHP, Koh JC, Lee JH. Association between admission body mass index and outcomes in critically ill children: A systematic review and meta-analysis. Clin Nutr (2021) 40:2772-83. doi: [10.1016/j.clnu.2021.04.010](https://doi.org/10.1016/j.clnu.2021.04.010)

7 Ventura JC, Hauschild DB, Barbosa E, Bresolin NL, Kawai K, Mehta NM, et al. Undernutrition at PICU admission is predictor of 60-day mortality and PICU length of stay in critically ill children. J Acad Nutr Diet (2020) 120:219-29. doi: [10.1016/j.jand.2019.06.250](https://doi.org/10.1016/j.jand.2019.06.250)

8 Nangalu R, Pooni PA, Bhargav S, Bains HS. Impact of malnutrition on pediatric risk of mortality score and outcome in Pediatric Intensive Care Unit. Indian J Crit Care Med (2016) 20:385-90. doi: [10.4103/0972-5229.186218](https://doi.org/10.4103/0972-5229.186218)

9 von Elm E, Altman DG, Egger M, Pocock SJ, Gøtzsche PC, Vandenbroucke JP, et al. The Strengthening the Reporting of Observational Studies in Epidemiology (STROBE) statement: Guidelines for reporting observational studies. Lancet (2007) 370:1453-7. doi: [10.1016/S0140-6736(07)61602-X](https://doi.org/10.1016/S0140-6736(07)61602-X)

10 VanderWeele TJ, Ding P. Sensitivity Analysis in Observational Research: Introducing the E-Value. Ann Intern Med (2017) 167(4):268-74. doi: 10.7326/M16-2607
